# Genome-wide Association Studies of over 30,000 Samples with Bone Mineral Density at Multiple Skeletal Sites and Its Clinical Relevance

**DOI:** 10.1093/gpbjnl/qzaf097

**Published:** 2025-11-05

**Authors:** Yu Qian, Jiangwei Xia, Pingyu Wang, Chao Xie, Hong-Li Lin, Gloria Hoi-Yee Li, Cheng-Da Yuan, Mo-Chang Qiu, Yi-Hu Fang, Chun-Fu Yu, Xiang-Chun Cai, Saber Khederzadeh, Pian-Pian Zhao, Meng-Yuan Yang, Jia-Dong Zhong, Xin Li, Peng-Lin Guan, Jia-Xuan Gu, Si-Rui Gai, Xiang-Jiao Yi, Jian-Guo Tao, Xiang Chen, Mao-Mao Miao, Guo-Bo Chen, Lin Xu, Shu-Yang Xie, Geng Tian, Hua Yue, Guangfei Li, Wenjin Xiao, David Karasik, Youjia Xu, Liu Yang, Ching-Lung Cheung, Fei Huang, Zhenlin Zhang, Hou-Feng Zheng

**Affiliations:** Suzhou Laboratory of Precision Health and Data Science, The Second Affiliated Hospital of Soochow University, Suzhou 215004, China; Institute of Health Data Science, Soochow University, Suzhou 215001, China; Institute of Basic Medical Sciences, Westlake Institute for Advanced Study and Westlake University, Hangzhou 310030, China; Department of Neurology, Xuanwu Hospital, National Center for Neurological Disorders, Capital Medical University, Beijing 100053, China; Binzhou Medical University, Yantai 264003, China; Institute of Science and Technology for Brain-Inspired Intelligence, Fudan University, Shanghai 200433, China; School of Public Health, The University of Hong Kong, Hong Kong Special Administrative Region 999077, China; Department of Health Technology and Informatics, Faculty of Health and Social Sciences, The Hong Kong Polytechnic University, Hong Kong Special Administrative Region 999077, China; Department of Dermatology, Hangzhou Hospital of Traditional Chinese Medicine, Hangzhou 310007, China; Jiangxi Medical College, Shangrao 334000, China; Jiangxi Medical College, Shangrao 334000, China; Department of Orthopedic Surgery, Shangrao Municipal Hospital, Shangrao 334000, China; Department of Orthopedic Surgery, Shangrao Municipal Hospital, Shangrao 334000, China; Institute of Basic Medical Sciences, Westlake Institute for Advanced Study and Westlake University, Hangzhou 310030, China; Institute of Basic Medical Sciences, Westlake Institute for Advanced Study and Westlake University, Hangzhou 310030, China; Suzhou Laboratory of Precision Health and Data Science, The Second Affiliated Hospital of Soochow University, Suzhou 215004, China; Institute of Health Data Science, Soochow University, Suzhou 215001, China; Suzhou Laboratory of Precision Health and Data Science, The Second Affiliated Hospital of Soochow University, Suzhou 215004, China; Institute of Basic Medical Sciences, Westlake Institute for Advanced Study and Westlake University, Hangzhou 310030, China; Suzhou Laboratory of Precision Health and Data Science, The Second Affiliated Hospital of Soochow University, Suzhou 215004, China; Institute of Basic Medical Sciences, Westlake Institute for Advanced Study and Westlake University, Hangzhou 310030, China; Suzhou Laboratory of Precision Health and Data Science, The Second Affiliated Hospital of Soochow University, Suzhou 215004, China; Institute of Basic Medical Sciences, Westlake Institute for Advanced Study and Westlake University, Hangzhou 310030, China; Suzhou Laboratory of Precision Health and Data Science, The Second Affiliated Hospital of Soochow University, Suzhou 215004, China; Institute of Basic Medical Sciences, Westlake Institute for Advanced Study and Westlake University, Hangzhou 310030, China; Suzhou Laboratory of Precision Health and Data Science, The Second Affiliated Hospital of Soochow University, Suzhou 215004, China; Institute of Basic Medical Sciences, Westlake Institute for Advanced Study and Westlake University, Hangzhou 310030, China; Institute of Basic Medical Sciences, Westlake Institute for Advanced Study and Westlake University, Hangzhou 310030, China; Institute of Basic Medical Sciences, Westlake Institute for Advanced Study and Westlake University, Hangzhou 310030, China; Institute of Basic Medical Sciences, Westlake Institute for Advanced Study and Westlake University, Hangzhou 310030, China; Institute of Basic Medical Sciences, Westlake Institute for Advanced Study and Westlake University, Hangzhou 310030, China; Clinical Research Institute, Zhejiang Provincial People’s Hospital, People’s Hospital of Hangzhou Medical College, Hangzhou 314408, China; Binzhou Medical University, Yantai 264003, China; Binzhou Medical University, Yantai 264003, China; Binzhou Medical University, Yantai 264003, China; Department of Osteoporosis and Bone Disease, Shanghai JiaoTong University Affiliated Six People’s Hospital, Shanghai 200233, China; Department of Orthopaedics, The Second Affiliated Hospital of Soochow University, Osteoporosis Research Institute of Soochow University, Suzhou 215005, China; Department of Orthopaedics, The Second Affiliated Hospital of Soochow University, Osteoporosis Research Institute of Soochow University, Suzhou 215005, China; Azrieli Faculty of Medicine, Bar-Ilan University, Safed 5290002, Israel; Department of Orthopaedics, The Second Affiliated Hospital of Soochow University, Osteoporosis Research Institute of Soochow University, Suzhou 215005, China; Institute of Orthopedic Surgery, Xijing Hospital, Fourth Military Medical University, Xi’an 710038, China; Department of Pharmacology and Pharmacy, Li Ka Shing Faculty of Medicine, The University of Hong Kong, Hong Kong Special Administrative Region 999077, China; Binzhou Medical University, Yantai 264003, China; Department of Osteoporosis and Bone Disease, Shanghai JiaoTong University Affiliated Six People’s Hospital, Shanghai 200233, China; Suzhou Laboratory of Precision Health and Data Science, The Second Affiliated Hospital of Soochow University, Suzhou 215004, China; Institute of Health Data Science, Soochow University, Suzhou 215001, China; Institute of Basic Medical Sciences, Westlake Institute for Advanced Study and Westlake University, Hangzhou 310030, China

**Keywords:** Bone mineral density, Drug target, Fracture, Genome-wide association study

## Abstract

The ultimate goal of a genome-wide association study (GWAS) is to translate its discoveries into clinical practice. To explore the clinical use of GWAS findings in the bone field, we conducted a GWAS of dual-energy X-ray absorptiometry (DXA)-derived bone mineral density (BMD) traits at 11 skeletal sites, within over 30,000 European individuals from the UK Biobank. A total of 91 unique and independent loci were identified for 11 DXA-derived BMD traits and fractures, including 5 novel loci (harboring the genes *ABCA1*, *CHSY1*, *CYP24A1*, *SWAP70*, and *PAX1*) for 6 BMD traits. These loci exhibited evidence of association in both males and females, which could serve as independent replication. We demonstrated that each polygenic risk score (PRS) was independently associated with fracture risk. Although incorporating multiple PRSs (*i.e.*, metaPRS) with clinical risk factors from the Fracture Risk Assessment Tool (FRAX) yielded the highest predictive performance, the improvement was modest in fracture prediction. Additionally, we uncovered genetic correlation and shared polygenicity between head BMD and intracranial aneurysm (IA). Finally, by integrating gene expression and GWAS datasets, we prioritized genes (*e.g.*, *ESR1* and *SREBF1*) encoding druggable human proteins along with their respective inhibitors/antagonists. In conclusion, this comprehensive investigation reveals a new genetic basis for BMD and its clinical relevance to fracture prediction. More importantly, it suggests that head BMD is genetically correlated with IA. The prioritization of genetically supported targets implies the potential repurposing of drugs [*e.g.*, omega-3 polyunsaturated fatty acid (PUFA) supplements] for the prevention of osteoporosis.

## Graphical abstract

**Figure:**
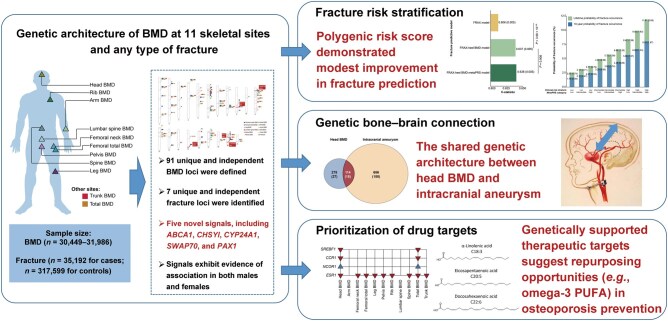

## Introduction

Osteoporosis, a systemic skeletal disease characterized by decreased bone mass and micro-structural damage [1,2], has a global prevalence of 18.3% [95% confidence interval (CI): 16.2%–20.7%] [3]. Bone mass can be assessed by 2-dimensional projectional scans with dual-energy X-ray absorptiometry (DXA), or other medical imaging tools, such as quantitative computed tomography (QCT) and quantitative ultrasound (QUS) [4]. Genome-wide association studies (GWASs) and meta-analyses have been carried out to explore the genetic factors for bone mineral density (BMD), osteoporosis, and fracture [1,2]. Early GWAS design only involved thousands of samples, and only several loci were identified [5,6]. Meta-analysis can enlarge sample size and statistical power, and lead to the identification of more loci [7,8]. However, the genetic summary data, instead of individual-level genotype data, from each cohort were meta-analyzed in the aforementioned studies. Recently, large-scale biobanks such as the UK Biobank enable access to individual-level genotype data in hundreds of thousands of samples, leading to the identification of hundreds of genetic loci for QUS-derived BMD [9,10].

Although GWASs have been successfully conducted in the past decade, the ultimate goal of genetic studies is to translate the discoveries into clinical practice. Previously, we have summarized the clinical use of GWAS findings in the bone field, such as disease prediction [1]. Lu et al. developed the genetically predicted speed of sound (SOS, a parameter measured by QUS) for individuals in the UK Biobank by common genetic variants through polygenic risk scores (PRSs) [11]. They demonstrated that this score provided modestly better fracture risk prediction than some clinical risk factors, such as smoking and corticosteroid use [11]. In addition, they suggested that adding rare variants did not demonstrate substantially improved predictive performance in a recent study [12]. The aforementioned studies used the SOS measurement in the training and testing datasets; however, it was not well correlated with BMD [13]. Another clinical relevance of GWAS findings is to infer the correlation between diseases [1]. Earlier efforts have uncovered numerous single-nucleotide polymorphisms (SNPs) exhibiting pleiotropic associations with BMD and other traits/diseases, such as birth weight [14], type 2 diabetes [15], and major depressive disorder [16]. Finally, incorporating genetic data in drug development is warranted to improve this process, because drugs with genetic support are more likely to succeed in clinical trials [17,18].

**Figure 1:**
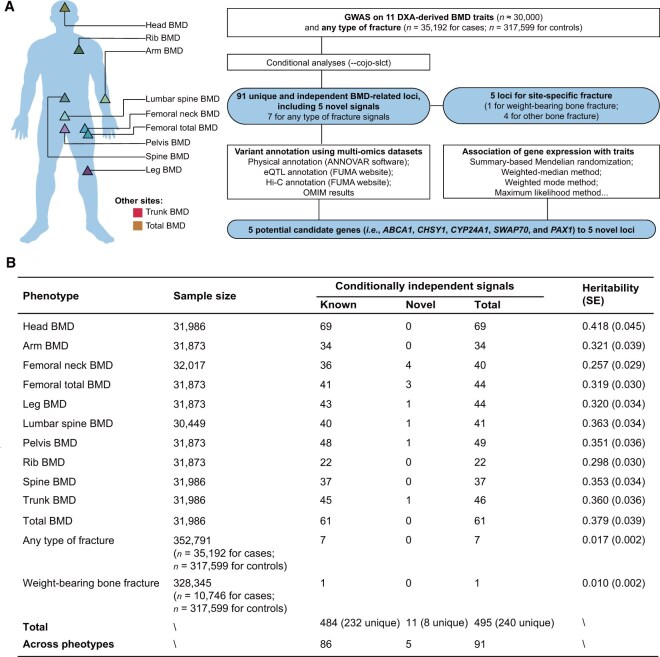
Genetic architecture of DXA-derived BMD at multiple skeletal sites **A**. Skeletal sites of 11 DXA-derived BMD traits and the GWAS design. **B**. Summary of loci identified for 11 BMD sites and fracture risk. BMD, bone mineral density; DXA, dual-energy X-ray absorptiometry; GWAS, genome-wide association study; SE, standard error.

Therefore, with the availability of DXA-derived BMD phenotypes and individual-level genotype data in the UK Biobank, there is an opportunity to conduct a GWAS at large scale using individual-level genotype data to investigate the genetic basis of BMD at 11 sites (arm, femoral total, femoral neck, head, leg, pelvis, lumbar spine, rib, spine, trunk, and total body) (**Figure 1**A) and fracture risk. We developed a “multi-BMD PRS” predictive model to improve genetic risk stratification for fracture. In addition, we estimated the shared genetic architecture of BMD with other common chronic diseases, including neurodegenerative, cardiovascular, and autoimmune diseases. Finally, we explored the potential effective and safe therapeutic targets for osteoporosis.

## Results

### Genetic architecture of BMD at multiple skeletal sites

We conducted the GWAS analyses for BMD at 11 anatomic sites (*i.e.*, arm, femoral total, femoral neck, head, leg, lumbar spine, pelvis, rib, spine, trunk, and total body) and for any type of fracture (Figure 1A) in males and females separately. For each BMD trait, we then conducted meta-analyses to combine the results from both genders (for BMD traits: *n* ≈ 30,000; for any type of fracture: *n* = 35,192 for cases, *n* = 317,599 for controls). All intercept values from the linkage disequilibrium (LD) score method were close to 1, revealing no obvious population stratification for either GWAS (Table S1). We observed that approximately 25.7%–41.8% of the variance in BMD and 1.7% of the variance in fracture risk could be explained by common variants across the genome (Figure 1B). We then conducted conditional analyses within phenotypes and identified 240 unique conditionally independent BMD signals (Figure 1B, Figures S1–S12; Table S2). After merging the physically overlapping signals across BMD phenotypes [*i.e.*, the distance between two conditionally independent single-nucleotide variants (SNVs) < 500 kb] into one locus, a total of 91 unique and independent BMD loci were defined (Figure 1B, Figure S1; Table S1). We identified seven unique and independent loci for any type of fracture, all overlapping with the aforementioned BMD signals (Figure 1B, Figures S1 and S13).

#### Five loci identified for DXA-derived BMD traits

Although previous GWASs have reported hundreds of loci, we still identified five loci for six BMD traits that have not been reported previously (Figure S1). Among these loci, the most pleiotropic locus resided between *ABCA1* and *SLC44A1* on chromosome 9 (Figure S1; **Table 1**, Tables S2 and S3). SNPs (rs1039406 and rs746100) around this locus were genome-wide significantly associated with five BMD sites, including the lumbar spine, femoral neck, femoral total, pelvis, and trunk (Figure S1; Table 1, Table S2). Whole-blood expression quantitative trait locus (eQTL) data from the Genotype-Tissue Expression (GTEx) project revealed that SNP rs746100 was also associated with the expression of *ABCA1* (*P* = 2.68 × 10^−5^) in tibial artery tissue (Figure S14; Table S4), and summary-based Mendelian randomization (SMR) results revealed that the genetically predicted higher *ABCA1* expression in whole blood was associated with higher BMD (Table 1, Table S5). Such an association was replicated with alternative Mendelian randomization (MR) methods (Table 1, Table S5). The second promising locus resided between *SWAP70* and *WEE1* on chromosome 11 with a lead SNP rs10840273, showing a genome-wide significant association with leg BMD (*P* = 4.52 × 10^−9^) (Figure S1;Table 1, Tables S2 and S3). Whole-blood eQTL data from eQTLGen identified that rs10840273 was associated with *SWAP70* expression (*P* = 2.42 × 10^−39^) (Figure S15; Table S4). Hi-C data from mesenchymal stem cells also detected a direct interaction of the associated region with the *SWAP70* gene [false discovery rate (FDR)-corrected *P*_interaction_ = 2.74 × 10^−109^] (Table S6). Furthermore, this lead SNP showed a genome-wide significant association with circulating SWAP70 protein (*P* = 6.94 × 10^−81^) (Figure S15). The results from multiple MR methods revealed that genetically predicted higher *SWAP70* expression and higher circulating SWAP70 protein level in whole blood were significantly associated with increased leg BMD (Table 1, Table S5).

**Table 1:** Genetic associations of novel loci with corresponding BMD traits in GWAS analyses

Exposure	Outcome	GWAS in males				GWAS in females				Sex-combined GWAS				Replicated in MR methods^*^
		Beta	SE	*P*	Number	Beta	SE	*P*	Number	Beta	SE	*P*	Number	
*ABCA1*														
rs1039406 (Chr9:107748958; allele T)	Lumbar spine BMD	0.041	0.012	3.75E−04	14,563	0.057	0.012	5.70E−06	12,964	0.049	0.009	1.21E−08	27,527	4
rs746100 (Chr9:107749051; allele C)	Femoral neck BMD	0.043	0.011	1.57E−04	15,349	0.058	0.012	1.77E−06	13,633	0.050	0.008	1.69E−09	28,982	4
	Femoral total BMD	0.049	0.011	1.74E−05	15,345	0.052	0.012	2.31E−05	13,593	0.050	0.008	1.64E−09	28,938	4
	Pelvis BMD	0.048	0.011	2.03E−05	15,314	0.049	0.012	5.67E−05	13,639	0.049	0.008	4.50E−09	28,953	3
	Trunk BMD	0.045	0.011	6.22E−05	15,314	0.050	0.012	3.74E−05	13,639	0.048	0.008	9.35E−09	28,953	4
*SWAP70*														
rs10840273 (Chr11:9642451; allele T)	Leg BMD	0.049	0.012	2.59E−05	15,326	0.050	0.012	4.44E−05	13,673	0.050	0.009	4.51E−09	28,999	4
*CHSY1*														
rs12916774 (Chr15:101710165; allele A)	Femoral neck BMD	0.063	0.014	9.72E−06	15,236	0.061	0.015	6.30E−05	13,523	0.062	0.010	2.44E−09	28,759	NA^#^
rs11630618 (Chr15:101710434; allele T)	Femoral total BMD	0.061	0.014	1.04E−05	15,227	0.050	0.015	7.25E−04	13,481	0.056	0.010	3.19E−08	28,708	NA^#^
*CYP24A1*														
rs35194449 (Chr20:52742047; allele C)	Femoral total BMD	0.069	0.014	1.35E−06	15,632	0.061	0.015	5.18E−05	13,848	0.065	0.010	3.10E−10	29,480	NA^#^
rs6013897 (Chr20:52742479: allele T)	Femoral neck BMD	0.068	0.014	1.77E−06	15,670	0.068	0.015	5.96E−06	13,915	0.068	0.010	4.56E−11	29,585	NA^#^
*PAX1*														
rs927059 (Chr20:21914194; allele T)	Femoral neck BMD	0.050	0.011	9.07E−06	15,651	0.042	0.012	4.89E−04	13,913	0.047	0.008	1.87E−08	2,9564	1^$^

*Note*: *, five MR methods (SMR, weighted median, maximum likelihood, weighted mode, and MR-Egger regression) were used to replicate the associations using eQTL data, and the number of methods used is shown in this column (see Table S5 for detailed information). ^#^, no instrumental variables are available for this gene. ^$^, only one instrumental variable is available for this gene. BMD, bone mineral density; GWAS, genome-wide association study; MR, Mendelian randomization; SMR, summary-based Mendelian randomization.

Another locus surrounding rs12916774 on chromosome 15 was associated with femoral neck and femoral total BMD (Figures S1 and S16; Table 1, Tables S2 and S3). The eQTL data and mesenchymal stem cell Hi-C data consistently supported *CHSY1* as a plausible candidate gene (*P* = 2.15 × 10^−53^ for *CHSY1* eQTL in whole blood from eQTLGen; FDR-corrected *P*_interaction_ = 1.72 × 10^−49^ for Hi-C data) (Table 1, Tables S4 and S6). The fourth locus (lead SNP: rs6013897) was located in the intergenic region between *CYP24A1* and *BCAS1* (Figures S1 and S17;Table 1, Tables S2 and S3). The mesenchymal stem cell Hi-C data detected a direct interaction of the associated region with the *CYP24A1* gene (FDR-corrected *P*_interaction_ = 8.04 × 10^−78^) (Table S6). We further prioritized *PAX1* as a potential candidate gene for rs927059, which was a lead SNP for femoral neck BMD (*P* = 1.87 × 10^−8^) (Figures S1 and S18; Table 1, Tables S2–S4). The positional and eQTL annotation results consistently supported *PAX1* as a candidate gene for rs927059 (*P* = 4.40 × 10^−6^ for *PAX1* eQTL in skeletal muscle tissue from GTEx) (Tables S3–S5). In summary, using multi-omics datasets, we prioritized five potential candidate genes (*i.e.*, *ABCA1*, *CHSY1*, *CYP24A1*, *SWAP70*, and *PAX1*) to five novel loci (Figure 1B, Figure S1; Tables S2–S4 and S6). The annotation results for other known loci are also shown in Tables S2–S4, S6, and S7.

#### Stratified analyses by sex and site

We reported BMD and fracture loci that exhibited evidence of association in both males and females (*P* < 0.05), providing independent replication (Table S8). Additionally, we identified one locus for weight-bearing bone fracture (rs2177470 on chromosome 7 near the *STARD3NL* gene, *P* = 8.83 × 10^−10^) (Figure 1A, Figure S19; Table S2). The A allele of this SNP was associated with a decreased risk of weight-bearing bone fracture (effect allele: A, odds ratio = 0.914, 95% CI = 0.888–0.941) (Table S2). This conditionally independent SNP was associated with other bone fractures with only nominal significance (*P* = 0.005) (Figure S20). However, this SNP showed a genome-wide significant association with lumbar spine BMD, indicating a positive effect of the A allele [beta = 0.059, standard error (SE) = 0.009, *P* = 2.77 × 10^−10^].

### PRS demonstrates modest improvement in fracture prediction

**Figure 2:**
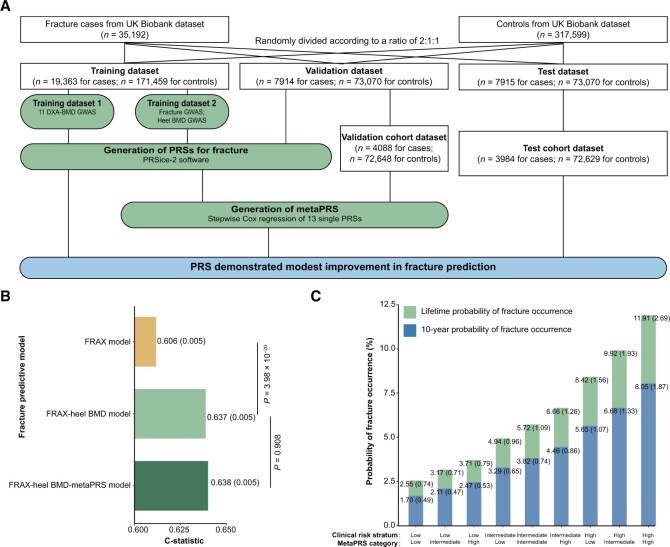
PRS demonstrates modest improvement in fracture prediction **A**. Study design. **B**. Fracture predictive results of three models. The FRAX model included clinical risk factors from FRAX tool (*i.e.*, sex, age, obesity, current smoking, current alcohol consumption, and glucocorticoid medicine use). For the FRAX-heel BMD model, heel BMD was integrated with the FRAX model. For the FRAX-heel BMD-metaPRA model, metaPRS was integrated into the FRAX-heel BMD model. Values in parentheses represent SD. **C**. Lifetime and 10-year probability of fracture occurrence across metaPRS categories within each clinical risk stratum. Values in parentheses represent SD. PRS, polygenic risk score; FRAX, Fracture Risk Assessment Tool; SD, standard deviation.

Based on effect sizes derived from GWASs of 11 DXA-BMD traits, heel BMD, and fracture in the training datasets, we selected SNPs that could achieve the best predictive PRS for fracture in the validation dataset. The number of selected SNPs ranged from 29 (for rib BMD) to 200,007 (for heel BMD) for different traits (**Figure 2**A; Table S9). After obtaining SNPs and the effect size for each trait, we calculated the corresponding PRS for each participant in the validation dataset. A metaPRS was generated by integrating these 13 individual PRSs using stepwise Cox regression in the validation cohort dataset, with estimates for each single PRS containing in the best-performing model (Figure 2A; Table S10). We then assessed the association between these PRSs and fracture risk in the test dataset. We found that the association of metaPRS with fracture incidence was largely independent of the traditional risk factors (*P*  = 3.77 × 10^−8^), and this metaPRS displayed the most prominent association [hazard ratio (HR): 1.106, 95% CI = 1.067–1.146], compared with single PRSs (Table S11).

By including only clinical factors from the Fracture Risk Assessment Tool (FRAX) model (*i.e.*, age, sex, obesity, smoking, alcohol use, and glucocorticoid use), we observed limited predictive performance of this model for any type of fracture [model 1: C-statistic = 0.606, standard deviation (SD) = 0.005] (Figure 2B). We found that adding heel BMD to model 1 increased the C-statistic to 0.637 (SD = 0.005) (model 2), with a *P* value for comparison of 3.98 × 10^−20^ (Figure 2B). However, the inclusion of metaPRS in model 2 did not substantially improve the C-statistic (*P*_comparison_ = 0.908), although the highest C-statistic was achieved (C-statistic = 0.638, SD = 0.005) (model 3) (Figure 2B). We further assessed how the interplay of the metaPRS and clinical risk factors impacts the fracture risk. Firstly, we found that the cumulative incidence for fracture events was 4.43% for individuals with low polygenic risk (bottom quintile of the metaPRS) and 7.79% among those with high polygenic risk (top quintile of the metaPRS), suggesting that metaPRS could stratify individuals into different trajectories of fracture risk (Figure S21). Although we observed significant gradients in the 10-year probability of fracture occurrence across metaPRS categories within each clinical risk stratum (Figure 2C), the clinical risk factors played a more important role in the stratification. For example, among participants with low clinical risk, the 10-year probability of fracture occurrence for those with high genetic risk (2.47%, SD = 0.53%) was yet lower than that for participants with intermediate clinical risk but low genetic risk (3.29%, SD = 0.65%) (Figure 2C). A similar association was also observed in the lifetime risk of incident fracture (Figure 2C).

#### Stratified analyses by age, population, and site

We further explored the heterogeneity in the prediction model performance across different age groups. We found that the prediction performance for any type of fracture, as measured by the C-index, increased with the age of the subgroup (*P*_heterogeneity_ = 1.35 × 10^−162^). The highest C-index was observed in participants aged ≥ 60 years (C-index = 0.637, SE = 0.006), followed by participants aged 50–60 years (C-index = 0.618, SE = 0.009) and participants aged < 50 years (C-index = 0.552, SE = 0.012). Additionally, we assessed the performance of these prediction models in predicting any type of fracture in non-European ancestry populations. Compared to the European population, the metaPRS showed lower predictive accuracy in non-European populations (C-index = 0.508, SE = 0.012). Similarly, clinical risk factors showed modest fracture prediction accuracy in non-European populations (C-index = 0.609, SE = 0.012). The combined model incorporating both clinical risk factors and metaPRS showed reduced predictive accuracy in non-European populations (C-index = 0.550, SE = 0.013). Finally, we generated a new prediction model focusing on the risk of fracture at the weight-bearing bone. We found that including only clinical factors from the FRAX model resulted in limited predictive performance (C-statistic = 0.654, SD = 0.008). Adding heel BMD to this FRAX model increased the C-statistic to 0.676. Incorporating metaPRS into the FRAX-heel BMD model resulted in the highest C-statistic (C-statistics = 0.679, SD = 0.007).

### Shared genetic architecture of head BMD and intracranial aneurysm

**Figure 3:**
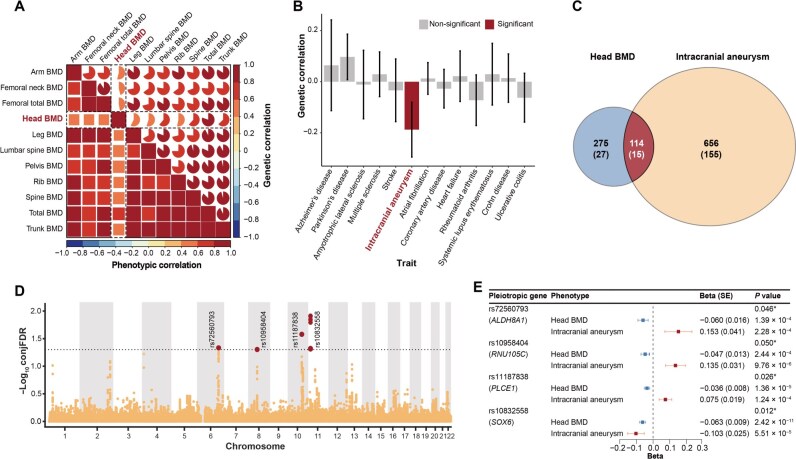
Shared genetic architecture of head BMD and intracranial aneurysm **A**. Heatmap showing genetic and phenotypic correlations between 11 DXA-BMD traits. **B**. Genetic correlations of head BMD with 13 common chronic diseases. **C**. Venn diagram showing shared variants between head BMD and intracranial aneurysm as well as unique variants per trait. Values in parentheses represent SD. **D**. Shared loci between head BMD and intracranial aneurysm. Common genetic variants jointly associated with head BMD and intracranial aneurysm at conjFDR < 0.05 are highlighted in red. **E**. Forest plot showing genetic association estimates for four variants jointly associated with head BMD and intracranial aneurysm.

We further estimated the shared genetic architecture of 11 DXA-BMD traits with other 13 common chronic diseases, including neurodegenerative, cardiovascular, and autoimmune diseases (Table S12). First, we tested the pair-wise correlation between the BMD traits. The weakest correlations were observed between head BMD and other BMD traits in both phenotypic and genetic correlation analyses, although all pairs exhibited statistically significant phenotypic correlations (**Figure 3**A). In the 143 BMD–disease pairs (11 DXA-BMD traits × 13 diseases), we only observed a statistically significant inverse genetic correlation of head BMD with intracranial aneurysm (IA) (*r*_g_ = –0.188, SE = 0.055, FDR-corrected *P* = 0.0096), while its genetic correlations with other 12 common chronic diseases were not significant (FDR-corrected *P* > 0.05) (Figure 3B; Table S12). Furthermore, no significant genetic correlation was observed for the remaining 10 DXA-BMD traits with IA (FDR-corrected *P* > 0.05) (Table S12). Compared to the specificity of observed genetic correlation, there was a similar MiXeR-estimated polygenic overlap between head BMD and IA. Additionally, 29.36% (*n* = 114, SD = 15) of the 389 head-BMD influencing variants were predicted to influence IA (Figure 3C; Table S13). By employing the conjFDR method, we identified four genomic loci jointly associated with head BMD and IA (Figure 3D–E; Table S14). Intriguingly, three of the four lead SNPs (rs72560793, rs10958404, and rs11187838) had the opposite effect direction, consistent with the moderate inverse genetic correlation between head BMD and IA (Figure 3E; Table S14). Notably, two of the four loci demonstrated strong evidence of colocalization (H4 > 0.5), suggesting the presence of shared causal variants between head BMD and IA (H4 = 0.809 for rs10832558 within *SOX6*; H4 = 0.581 for rs11187838 within *PLCE1*) (Table S15). Genes mapped to these shared loci were enriched for biological processes and cellular components related to the skeletal systems (*e.g.*, positive regulation of chondrocyte differentiation) and vascular smooth muscle (*i.e.*, regulation of Ras protein signal transduction) (Table S16). The bidirectional MR analyses did not reveal evidence for the potential causality (all *P* > 0.05) (Table S17).

### Prioritization of drug targets

**Figure 4:**
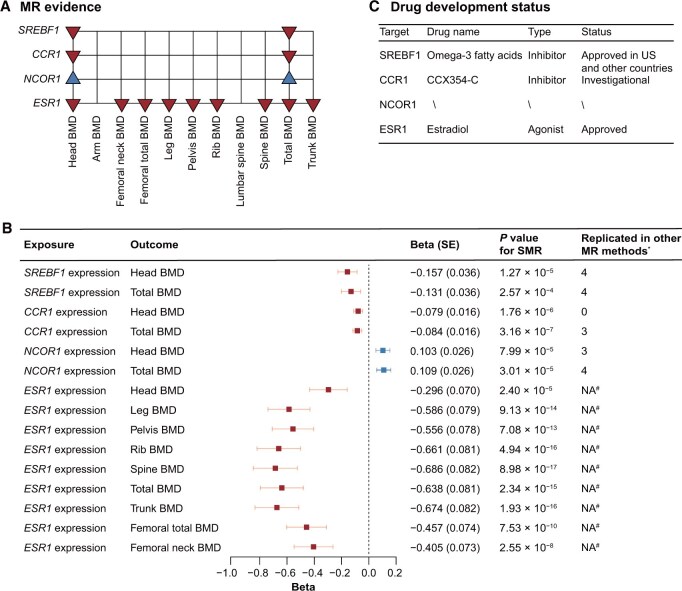
Prioritization of drug targets **A**. Four genes with MR evidence. Red downward triangle indicates that genetically predicted expression level of this gene is negatively correlated with BMD, while blue upward triangle indicates a positive association for BMD. **B**. Forest plot showing associations of genetically predicted expression of *SREBF1*, *CCR1*, *NCOR1*, and *ESR1* with BMD, based on SMR analyses. *, four other MR methods (weighted median, maximum likelihood, weighted mode, and MR-Egger regression) were used to replicate the associations, and the number of replications in these MR methods is shown in this column. ^#^, no instrumental variables are available for this gene. **C**. Drug development status of SREBF1, CCR1, NCOR1, and ESR1. MR, Mendelian randomization; SMR, summary-based Mendelian randomization.

Subsequently, we integrated the druggable genome, gene expression, and GWAS datasets to identify genetically supported potential therapeutic targets for osteoporosis, emulating exposure to corresponding medications. Utilizing drug target information from the ChEMBL database (release 29), we included a total of 3329 druggable genes for subsequent analyses. Next, we employed eQTL data from muscle (791 druggable genes), tibial artery (917 druggable genes), and whole blood (845 druggable genes from GTEx; 2104 druggable genes from eQTLGen) to test associations with BMD through an MR approach. We found 15 genes whose genetically predicted expression was significantly associated with DXA-BMD (FDR-corrected *P* < 0.05) (Table S5). Among these, four genes (*CCR1*, *ESR1*, *NCOR1*, and *SREBF1*) showed consistent directional associations with at least two DXA-BMD traits, providing robust MR evidence for these genes (**Figure 4**A and B; Table S5). For these four genes, genetically predicted *ESR1* expression showed negative associations with nine DXA-BMD traits (Figure 4A and B; Table S5). Positive associations were observed for *NCOR1* expression with head BMD and total BMD, while negative associations were found for *SREBF1* and *CCR1* expression (Figure 4A and B; Table S5). We then applied alternative MR methods and found that most SMR findings were replicated in at least two MR methods (Figure 4A and B; Table S5). To assess whether the genetic associations of these genes with DXA-BMD traits shared a common causal variant, we conducted colocalization analyses using the coloc and SharedPro methods. We discovered that whole-blood eQTLs for all four genes (*i.e.*, *SREBF1*, *NCOR1*, *ESR1*, and *CCR1*) colocalized with DXA-BMD loci (H4 > 0.5), reinforcing the evidence for these genes as drug targets for DXA-BMD (Tables S18 and S19). Considering the observed negative associations of *SEEBF1* and *CCR1* expression with BMD (Figure 4A and B; Table S5), existing inhibitors/antagonists that have been approved or under investigation represent possible repurposing opportunities for osteoporosis treatment. Specifically, SEBF1 could be targeted using doconexent (inhibitor) and omega-3 fatty acids (inhibitor), while CCR1 could be targeted using CCX354-C (antagonist) (Figure 4C).

## Discussion

In this study, we first conducted large-scale GWASs of DXA-BMD at 11 skeletal sites, and identified 91 unique and independent loci associated with at least one trait, including five previously unreported BMD loci (*i.e.*, *ABCA1*, *CHSY1*, *CYP24A1*, *SWAP70*, and *PAX1*) for six BMD traits. These novel loci exhibited evidence of association in both males and females, which could serve as independent replication. Additionally, the incorporation of multiple PRSs (metaPRS) with the clinical risk factors exhibited the highest predictive performance; however, the improvement was modest in fracture prediction. We further estimated the shared genetic architecture of DXA-BMD at 11 sites with other common chronic diseases, including neurodegenerative, cardiovascular, and autoimmune diseases, and only uncovered genetic correlation and shared polygenicity between head BMD and IA. Finally, by integrating gene expression and GWAS datasets, we prioritized genes (*i.e.*, *ESR1*, *SREBF1*, *CCR1*, and *NCOR1*) encoding druggable human proteins along with their respective inhibitors/antagonists.

Although previous GWAS have identified hundreds of association signals [1,2], we reported five novel loci in this study. The lead SNPs in these loci improved at least two orders of magnitude of significance compared to the most significant SNP within the corresponding region (reported SNP position ± 250 kb) in any previous BMD GWASs. For example, in our study, the locus near *ABCA1* (lead SNP: rs746100) was associated with five BMD traits (lumbar spine, femoral neck, femoral total, pelvis, and trunk), with the smallest *P* value of 1.64 × 10^−9^. In contrast, a previous meta-analysis of GWAS with a comparable sample size (∼ 30,000) reported that SNP rs1831554 within this locus showed marginal significance for femoral neck BMD (*P* = 9.94 × 10^−5^) and lumbar spine BMD (*P* = 1.41 × 10^−4^) [8]. The pair-wise LD of the two lead SNPs was 0.0005. In our study, we used the individual-level genotype data from ∼ 30,000 samples; the sample size was comparable to the GWAS meta-analysis of summary statistic data [8], but the association significance improved substantially. It has been suggested that an association analysis performed with individual-level genotype data could enable a more comprehensive power to control various factors, such as population structure, covariates, and phenotype definitions [19]. Another example was the locus near *CHSY1*. This locus showed marginal significance (*P* = 2.30 × 10^−5^ for rs3784491) in the largest-scale GWAS to date for QUS-derived heel BMD [10], with a sample size more than ten times that of our study. However, the SNP rs12916774, in very low LD with rs3784491 (LD *r*^2^ = 0.005), was found to be genome-wide significantly associated with femoral neck BMD in our study (*P* = 2.14 × 10^−9^). It should be noted that QUS-derived BMD primarily reflected the bone mass at the heel calcaneus and exhibited limited correlation (0.50–0.65) with DXA-derived BMD at the spine and hip [20]. Additionally, we confirmed the *ZIC1*/*ZIC4* locus for head BMD (*P* = 2.19 × 10^−8^) which was reported in a recent GWAS meta-analysis [21].

One of the potential applications of genetic data is disease prediction [1]. Lu et al. calculated the genetically predicted SOS (measured by QUS at the heel) for individuals in the UK Biobank and assessed the predictive performance of this score [11]. In this study, we used three independent datasets and generated PRSs for DXA-derived BMD at multiple skeletal sites. Our results indicated that PRSs had robust associations with incident fracture, even after adjusting for the related clinical risk factors such as age, sex, obesity, smoking, alcohol, glucocorticoid use, and BMD, suggesting an independent contribution to the susceptibility of fracture. Considering that using genetically correlated phenotypes can increase the effective sample size [22], we further built metaPRS by combining multiple PRSs for DXA-BMD, heel BMD, and fracture to evaluate the potential of PRSs on fracture prediction. As expected, metaPRS showed a larger effect size on fracture risk than fracture PRS. This improvement could be attributed to that the genetic component of this metaPRS captured the majority of the genetic basis of fracture. At baseline, we included the FRAX factors [23] in the prediction model, and only limited predictive performance was observed as before [24]. We observed an increase in the C-statistic when incorporating heel BMD into the FRAX model. However, the addition of various PRSs to the FRAX-heel BMD model did not substantially improve the C-statistic, suggesting that the predictive performance of PRS did not perform as well as the heel BMD measurement itself. Additionally, the probability of fracture occurrence for those with low clinical risk and high genetic risk was yet lower than the participants with intermediate clinical risk but low genetic risk, suggesting that the clinical risk factors play a more important role in the stratification. Consistently, Lu et al. also suggested that the predictive performance of PRS would not outperform BMD [11].

Clinically, IA is characterized by a bulge or distention of an artery in the brain due to weakness and inelasticity of the vessel wall [25]. The disruption of the extracellular matrix (ECM) has been proposed as a contributing factor in the pathophysiology of IA [26]. The ECM is also a salient feature of bone tissue. Bone ECM, containing minerals deposited on highly crosslinked collagen fibrils, dynamically interacts with osteoblasts and osteoclasts to regulate the process of bone regeneration [27]. Given the shared histological basis of bone and vessel, the genetic correlation analysis in this study suggested that higher head BMD would be associated with a lower risk of IA. This genetic association was supported by an epidemiological study that the IA risk was increased in patients with BMD in the middle and lower tertiles compared with those with BMD in the higher tertile [28]. However, the MR analyses did not support the potential causality between IA and head BMD, suggesting that the observed association may be due to shared etiological factors rather than direct causality [29]. Additionally, based on conditional/conjunctional FDR analyses, we identified four shared signals, emphasizing the pleiotropic effect underlying BMD and IA. Two of them demonstrated evidence of colocalization (rs10832558 within *SOX6* and rs11187838 within *PLCE1*). The SNP rs10832558 showed the same effect direction for head BMD and IA, which was not consistent with the inverse genetic correlation. Here, we highlighted the SNP rs11187838 shared by BMD and IA with the opposite effect direction, which had not been detected by both previous single-trait analyses. This SNP was mapped to the *PLCE1* gene, encoding the enzyme phospholipase C epsilon-1. This enzyme could stimulate the Ras and mitogen-activated protein kinase (MAPK) signaling pathway through the regulation of heterotrimeric G protein Galpha [30]. Ras signaling stimulated the proliferation of immature osteoprogenitor cells to increase the number of osteoblastic descendants in a cell-autonomous manner [31]. Additionally, the activation of Ras/MAPK signaling could stimulate the migration and proliferation of vascular smooth muscle cells through fibronectin [32]. These synthetic vascular smooth muscle cells could secrete large amounts of ECM components, including collagen, elastin, and matrix metalloproteinase, causing vascular ECM remodeling [33]. All these results suggested that *PLCE1* might play important roles in the shared polygenicity between BMD and IA. Finally, we did not observe genetic correlations between BMD and other diseases in our study. As the global genetic correlation represented the average of genome-wide shared association, the non-significant global correlation might be due to opposing directions at different genomic regions [34].

Several pharmacological agents are available to osteoporosis patients, either by reducing bone resorption, such as bisphosphonate and denosumab, or by stimulating bone formation, such as teriparatide and abaloparatide [35]. The fruitful GWAS discoveries in the bone field have proven useful in identifying compounds suitable for drug repurposing [36]. One possible approach is to use genetic variants associated with the expression level of a gene encoding a druggable human protein to proxy lifelong exposure to a medication targeting corresponding gene production [37,38]. This MR approach could mimic a randomized controlled trial to cost-effectively predict the treatment response of a drug [37,38]. In this study, by using GWAS data and eQTL data, we prioritized several drug targets for osteoporosis, including ESR1 and SREBF1. Estrogen hormone therapy, targeting ESR1 protein, was an old-fashioned treatment for osteoporosis rarely used nowadays because of adverse side effects such as cardiovascular conditions and cancer [39]. SREBF1, which we highlighted here, was the target of doconexent (a high-docosahexaenoic acid supplement) and omega-3 fatty acids. Daily marine omega‐3 supplementation has been widely recommended in the prevention of adverse coronary events [40,41]. However, the effect of this kind of fatty acid on bone health is controversial. For example, a meta-analysis of 23 randomized controlled trials did not show any significant effect of omega-3 polyunsaturated fatty acid (PUFA) supplementation on BMD in any body part [42]. Nevertheless, subgroup analyses revealed that the impact of omega-3 PUFA supplementation on BMD varied across different regions [42]. Specifically, individuals from Eastern countries exhibited higher BMD at the lumbar spine and femoral neck following omega-3 PUFA supplementation, in comparison to individuals from Western countries [42]. However, another systematic review and meta-analysis of randomized controlled trials suggested that omega-3 PUFAs may have a beneficial effect on bone health, particularly in postmenopausal women [43]. Fish oil supplements, which are rich in marine-derived long-chain omega-3 PUFAs, have also been associated with a reduced risk of fractures in several studies [44–46]. These inconsistent findings may be attributed to measurement errors in dietary omega-3 PUFA intake, potential confounding by other nutrients in the same food sources, and differences in cooking methods. In our study, we revealed a negative association between *SREBF1* expression and BMD. Previous studies have suggested that supplementation with omega-3 PUFAs downregulates SREBF1 [47,48], and that the decreased *SREBF1* expression could inhibit osteoclast formation and bone resorption activity by suppression NF-κB signaling [49]. Therefore, we hypothesized that omega-3 PUFA supplementation might be effective for the prevention of osteoporosis. For the CCR1 antagonist, BMS-817399 failed in a Phase 2, double-blind, placebo-controlled clinical trial [50], while another CCR1 antagonist (CCX354-C) has shown a good safety and tolerability profile and evidence of clinical activity in rheumatoid arthritis in Phase II trials (NCT01242917) [51]. A previous animal study has shown that the activation of CCR1 leads to the formation of osteolytic lesions through the regulation of CCL3 [52].

Although we did comprehensive analyses in this study, we would like to clarify several limitations. First, the prediction model was primarily generated in individuals of European ancestry from the UK Biobank, which limits its generalizability to other ancestral groups. Thanks to several large-scale genomic initiatives aimed at including diverse populations, such as the Trans-Omics for Precision Medicine [53] and the GenomeAsia Project [54], optimal performance and applicability across diverse populations might be ensured. Our group has also initiated the China Precision BioBank project (CPBB; http://www.cpbb.cn) to investigate the variant–BMD associations and to improve the ancestry-specific prediction model for the Chinese population [55]. Second, we did not present a prediction model for specific fracture sites, because the sample size for each site is small. To balance statistical power and specific sites in the analysis, we categorized the fractures into weight-bearing bones (*e.g.*, hip and vertebral fractures) and other bones (*e.g.*, skull and wrist fractures) following our previous work [15]. Third, the FRAX-heel BMD model in this study represents a simplified implementation rather than the complete FRAX calculation. The FRAX tool includes more complicated, non-linear, and/or interaction effects [56], which are unlikely to be captured by multivariable linear models.

In conclusion, we conducted large-scale GWASs of DXA-derived BMD traits and identified novel signals that are likely to provide new insights into the biological mechanism of osteoporosis. We demonstrated that although PRSs were independently associated with fracture risk, their predictive performance improved modestly compared to the clinical risk factors. Additionally, we uncovered a genetic correlation between head BMD and IA, and the jointly associated genes such as *PLCE1* might play important roles in the shared genetic basis. Finally, the prioritization of genetically supported targets implied the potential repurposing of drugs (*e.g.*, omega-3 PUFA supplements targeting SREBF1) for the prevention of osteoporosis.

## Materials and methods

### Source of phenotype data and quality control

As in our previous studies [57–59], the individual-level data from the UK Biobank (Application No. 41376) were used for discovery analyses. The UK Biobank is a cohort of approximately 500,000 participants aged 40–69 years, of which 487,409 were genotyped with the UK Biobank Axiom Array or the UK UKBiLEVE Array and then imputed by the 1000 Genomes Project (Phase 3) reference panel [60,61]. In this study, we extracted BMD traits measured by DXA from 11 anatomical sites (*i.e.*, arm, femoral total, femoral neck, head, leg, lumbar spine, pelvis, rib, spine, trunk, and total body) and fracture as phenotypes (Figure 1A; Table S20). Fracture cases were defined as participants diagnosed with any fracture (excluding fractures caused by known primary diseases or diseases that might affect bone health) (Table S20). We categorized fractures into weight-bearing bones (*e.g.*, hip and vertebral fractures) and other bones (*e.g.*, skull and wrist fractures) following our previous work [15]. To minimize population stratification bias, we further excluded participants who were not of European ancestry (Table S20) and those who had a kinship with any participants (the kinship threshold for related individuals was set at 0.0442). For quality control (QC) of the genotype data, variants were excluded if they met any of the following criteria: minor allele frequency (MAF) < 0.01, imputation info score < 0.3, missing genotype rate > 0.05, or *P* value for Hardy–Weinberg equilibrium test < 1 × 10^−6^. Given that the UK Biobank has stringent QC procedures in place, we did not observe any BMD values of zero. Therefore, no additional QC for outliers was conducted. A total of 5,996,792 imputed variants (located on autosomal chromosomes) and approximately 30,000 participants (Figure 1A; Table 1) remained for BMD GWAS, 352,791 participants (*n* = 35,192 for cases; *n* = 317,599 for controls) for fracture GWAS, and 328,345 participants (*n* = 10,746 cases; *n* = 317,599 controls) for weight-bearing bone fracture GWAS (Figure 1A; Table 1).

### Genetic association analysis of BMD and fracture

To identify the genetic variants associated with BMD at a genome-wide significant level (*P* ≤ 5 × 10^−8^), we conducted GWAS analyses on BMD traits at 11 skeletal sites. For BMD at each site, the values (g/cm^2^) were stratified by sex, and then adjusted for age, age^2^, weight, menopause status (only for females), and the first five principal components using linear regression. The standardized residuals (mean = 0 and SD = 1) in males and females (*i.e.*, standardized BMD) were used in the GWAS analyses. The associations between genetic variants and phenotypes (*i.e.*, standardized BMD at 11 skeletal sites) were analyzed using PLINK (v1.9) [62]. We also analyzed the associations between genetic variants and risk of any type of fracture or weight-bearing bone fracture, adjusting for age, weight, and the first five principal components using PLINK (v1.9), separately in male and female populations. We then combined the summary statistics of the two sexes using an inverse-variance weighted fixed effects meta-analysis implemented in METAL [63]. The lead SNPs of novel loci from sex-stratified GWAS analyses with *P* < 0.05 were considered replicated.

### Identification of statistically independent and novel loci

The conditionally independent signals for each BMD trait (between-sex meta-analysis) were defined using the conditional and joint (COJO; gcta --cojo-slct) analysis [64]. An LD reference panel was constructed using 10,000 randomly selected, unrelated white British individuals from the UK Biobank. The conditionally independent SNV for each signal was defined as the SNV with *P <* 5 ×10^−8^ in both the original GWAS and COJO analyses. Among these independent signals, the association was classified as a “novel” signal if all SNPs within one signal (conditionally independent SNV ± 250 kb) have not been reported to be significantly associated with BMD (*P* > 1 × 10^−6^) in previous BMD GWAS analyses [8,10,21]. Across 11 BMD traits, the identified conditionally independent significant SNVs were merged into one locus if they were closely located to each other (< 500 kb), leaving the SNP with the smallest *P* value as the lead SNP. The pleiotropic genomic locus was defined as a genomic locus containing multiple conditionally independent signals for different BMD traits.

### Variant annotation

We then performed functional annotation of the conditionally independent significant SNVs using ANNOVAR [65] and Functional Mapping and Annotation of Genome-wide Association Studies (FUMA; https://fuma.ctglab.nl/) [66], as well as the Online Mendelian Inheritance in Man database (OMIM; http://omim.org/) [67]. Specifically, these SNVs were first physically annotated using ANNOVAR. Subsequently, based on the FUMA platform, we further obtained the eQTL and chromatin interaction annotation results. The eQTL datasets were sourced from the eQTLGen Consortium and from five tissue types (*i.e.*, tibial artery, whole blood, and skeletal muscle) in the GTEx project (v8), and the long-range chromatin interaction (Hi-C) data were obtained from the GSE87112 dataset (mesenchymal stem cell). Additionally, we performed the gene map search in the OMIM dataset using ‘(OSTEOPOROSIS OR “bone fragility” OR “fragile bones” OR “bone mineral density”)’ to obtain a gene list for BMD phenotype. For each physically annotated gene, we collected corresponding evidence codes from the aforementioned datasets (p for physical annotation; e for eQTL annotation; h for HiC annotation; o for OMIM results).

### Integrating PRS with clinical risk score for fracture risk stratification

#### Training, validation, and test datasets

We evaluated the potential clinical utility of PRSs for fracture incidence combined with traditional clinical risk factors. For this purpose, two training datasets were set in the analyses for DXA-derived BMD (training dataset 1) and heel BMD/fracture (training dataset 2), respectively (Figure 2A). Training dataset 1 was derived from the aforementioned DXA-derived BMD GWAS. Additionally, all fracture cases (*n* = 35,192) and controls (*n* = 317,599) from the UK Biobank were randomly divided into three subsets at a 2:1:1 ratio: the training dataset 2 (*n* = 171,459 for controls, *n* = 19,363 for cases), the validation dataset (*n* = 73,070 for controls, *n* = 7914 for cases), and the test dataset (*n* = 73,070 for controls, *n* = 7915 for cases). Following this, GWAS analyses for both heel BMD and fracture were performed on training dataset 2 utilizing the aforementioned GWAS pipeline.

#### Construction of PRSs

Based on GWAS summary statistics from 13 traits (*i.e.*, 11 DXA-derived BMD traits, heel BMD, and fracture) in the training datasets, we used the clumping and threshold approach in PRSice-2 [68] to construct PRSs for fracture in the validation dataset. The best predictive PRSs were assessed for transferability and predictivity through the *P* values and Nagelkerke *R*^2^ in a logistic model implemented in PRSice-2 [69], which corrected for age, sex, weight, and population stratification (the first five principal components). After obtaining the *P* value threshold for the best predictive PRS from the validation dataset, we calculated the corresponding PRS for each participant in the test dataset.

#### Construction of metaPRS

To construct a combined PRS (*i.e.*, metaPRS), we first removed 4248 participants with a fracture history at baseline to generate a validation cohort dataset (*n* = 72,648 controls; *n* = 4088 cases) (Figure 2A). Based on this validation cohort dataset, we included all 13 PRSs to conduct stepwise Cox regression, which could automatically select a reduced number of predictor variables for building the best-performing Cox regression model. The metaPRS was subsequently calculated as a weighted sum of the selected PRSs, with weights defined by their respective beta coefficients from the stepwise Cox regression.

#### Performance assessment in predicting risk of any type of fracture

In this analysis, we further removed participants with a fracture history at baseline from the test dataset, leaving 76,613 participants as the test cohort dataset for fracture prediction (*n* = 72,629 controls; *n* = 3984 cases) (Figure 2A). We first constructed a basic FRAX-hell BMD model, incorporating the clinical risk factors from the FRAX tool [*i.e.*, sex (categorical: male and female), age (continuous: year), obesity (categorical: 1st, BMI ≤ 20; 2nd, 20 < BMI ≤ 25; 3rd, 25 < BMI ≤ 30; 4th, 30 < BMI ≤ 35; 5th, 35 < BMI ≤ 40; 6th, 40 < BMI ≤ 45; 7th, BMI > 45), current smoking (categorical: yes and no), current alcohol consumption (categorical: yes and no), and glucocorticoid medicine use (categorical: yes and no)] and heel BMD (Table S20). Using Cox regression for fracture, we obtained predicted values based on this basic FRAX-heel BMD model in the test cohort dataset. We then employed the C-statistic as a quantitative measure to evaluate the accuracy of the basic FRAX-heel BMD model using these predicted values in the same dataset. Additionally, we quantify the variations in discriminative power when integrating various PRSs into the basic FRAX-heel BMD model (FRAX-heel BMD-PRS model). Specifically, for each type of PRS (*i.e.*, heel BMD PRS, 11 DXA-BMD site-specific PRSs, fracture PRS, and metaPRS), we performed a multiple Cox regression for fracture, adjusting for age, sex, obesity, smoking, alcohol, glucocorticoid medicine use, heel BMD, and population stratification (the first five principal components). Based on these predicted values, C-statistic was used to estimate the improvement in discrimination and reclassification after adding each PRS to the basic FRAX-heel BMD model. Furthermore, to visualize the cumulative fracture incidence across polygenic risk categories [*i.e.*, low (bottom quartile), intermediate (middle two quartiles), and high (top quartile) stratified by metaPRS], we employed the survminer R package in the test cohort dataset consisting of time-to-fracture information and corresponding fracture events as well as polygenic risk categories. We also utilized the *cuminc* function to plot the cumulative incidence curves. Based on the *survfit* function from the survcomp R package, we estimated the 10-year absolute fracture risk and assessed the interplay between the metaPRS and the clinical risk score (from the basic FRAX-heel BMD model) in influencing the risk of fracture. Additionally, we explored the heterogeneity in model performance across three subgroups defined by baseline age: Group A (participants aged ≥ 60 years; 2151 cases for any type of fracture and 30,811 controls), Group B (participants aged 50–60 years; 1100 cases for any type of fracture and 23,513 controls), and Group C (participants aged < 50 years; 600 cases for any type of fracture and 16,618 controls). We also assessed the performance of these prediction models in predicting any-type fracture risk in non-European ancestry. The detailed definition of participants of non-European ancestry is listed in Table S20. We further generated new prediction models focused on the risk of fracture at weight-bearing bone sites using the same protocol.

### Shared genetic basis of BMD and common chronic diseases

#### Genetic correlation and polygenic overlap

In this study, we first estimated the phenotypic correlation between 11 DXA-derived BMD traits (*i.e.*, arm, femoral neck, femoral total, head, leg, lumbar spine, pelvis, rib, spine, total body, and trunk BMD) using Spearman correlation. We then assessed their genetic correlations using the GCTA software, considering the sample overlap. Additionally, we performed linkage disequilibrium score regression (LDSC) analyses [69] based on the 1000 Genomes Project European panel, to assess the genome-wide genetic correlations (*r*_g_) between 11 DXA-BMD traits and 13 selected common chronic diseases, including neurodegenerative diseases (Alzheimer’s disease, Parkinson’s disease, amyotrophic lateral sclerosis, and multiple sclerosis) [70–73], cardiovascular diseases (stroke, IA, atrial fibrillation, coronary artery disease, and heart failure) [74–78], and autoimmune diseases (rheumatoid arthritis, systemic lupus erythematosus, and inflammatory bowel disease) [79–81]. For BMD phenotypes with statistically significant genetic correlations, we applied the bivariate causal mixture model (MiXeR) to quantify the polygenic overlap between BMD and selected chronic diseases beyond genetic correlation [82]. For a pair of phenotypes, MiXeR estimated the number of trait-influencing SNPs (*i.e.*, SNPs with effects on the disease not inducted by LD) for each trait and the number of shared trait-influencing SNPs based on a bivariate Gaussian mixture model [82]. Additionally, we performed bidirectional MR analyses and sensitivity analyses to assess the potential causality between head BMD and IA (see File S1 for detailed methods).

#### Discovery of shared risk loci

To discover pleiotropic genetic variants, we performed conditional/conjunctional false discovery rate (condFDR/conjFDR) analyses using genetic summary statistics. We restricted our analysis to BMD phenotypes with evidence to support shared genetic architecture with common chronic diseases. Based on an empirical Bayesian statistical framework, in the condFDR method, the association between variant and secondary phenotype was used to re-rank the test statistics and re-calculate the association of this variant with the primary phenotype [83,84]. The conjFDR is defined as the maximum of two condFDR values, which provides a conservative estimate of the posterior probability that a genetic variant is associated with either trait [83,85]. In this study, the shared genetic variants were defined as variants with conjFDR < 0.05. For these identified risk loci with shared effects, we further used the coloc R package to determine whether the association signals for DXA-derived BMD and common chronic diseases would colocalize within the shared loci. After extracting genetic association estimates for variants within 250 kb of the lead SNP, the probability of H4 that the two traits share one causal variant was calculated. Loci with a probability of H4 > 0.5 were considered colocalized [86].

### Genetic-driven prioritization of drug targets

The therapeutic target lists were obtained from the ChEMBL database (release 29), which curates drug information from multiple sources (*e.g.*, United States Adopted Name applications, ClinicalTrials.gov, and FDA Orange Book) [87]. Specifically, based on the target search results, only proteins with values of activity term ≤ 100 and originating from *Homo sapiens* were retained. This resulted in a total of 3329 unique druggable genes that encode human target proteins with ENSG IDs for approved drugs or clinical candidates for subsequent analyses (Table S21). We performed bioinformatic analyses (*i.e.*, SMR and colocalization) to prioritize putative druggable genes for BMD treatment. First, we assessed whether the potential genetically regulated expression levels of these druggable genes were associated with DXA-BMD using SMR [88]. In SMR analyses, the genetic variants were used as instrumental variables to link the exposure of interest (*i.e.*, the expression level of the candidate gene) to the outcome (*i.e.*, DXA-BMD). Instrumental variables were extracted from *cis*-eQTLs in three tissues (muscle, tibial artery, and whole blood) from the GTEx project (v8) [89] and in whole blood from the eQTLGen consortium [90]. Linkage clumping was conducted based on default protocols (File S1). For each DXA-BMD phenotype, the SMR results of the druggable genes with FDR-corrected significance (FDR-corrected *P*_SMR_ < 0.05 and *P*_HEIDI_ > 0.05) were retained. Among genes with SMR evidence, we conducted additional MR analyses, including weighted median, maximum likelihood, weighted mode, and MR-Egger regression, to evaluate the robustness of these findings (File S1). Furthermore, we assessed whether the eQTL and DXA-BMD association signals would colocalize at shared loci (*i.e.*, the probability of H4). Specifically, after extracting genetic association estimates of eQTL and DXA-BMD traits with variants within 250 kb of the lead SNP, colocalization analyses were performed. The genes with a probability of H4 > 0.5 were considered colocalized [91]. Additionally, we applied the SharedPro method to improve the statistical power, based on a relaxed assumption [92]. The drug information of genes with SMR evidence was obtained from the GeneCards website (https://www.genecards.org), which collected information from DrugBank, ApexBio, DGIdb, ClinicalTrials.gov, and/or PharmGKB.

## Ethical statement

Ethics approval for the UK Biobank research was obtained from the North West Multicentre Research Ethical Committee (Approval No. 21/NW/0157), and all participants provided informed consent.

## Supplementary Material

qzaf097_Supplementary_Data

## Data Availability

The GWAS summary-level data on 11 DXA-BMD traits and fracture risk have been deposited in the Genome Variation Map (GVM) at the National Genomics Data Center (NGDC), China National Center for Bioinformation (CNCB) (GVM: GVP000082; BioProject: PRJCA050153), which are publicly accessible at https://bigd.big.ac.cn/gvm/getProjectDetail?Project=GVP000082. The data have also been deposited in the China Precision BioBank (CPBB) (https://cpbb.cn/resources/BMDGWASeuro), which is developed from the Westlake BioBank for Chinese (WBBC) Project [55,93–95].

## CRediT author statement

**Yu Qian:** Writing – original draft, Writing – review & editing, Formal analysis. **Jiangwei Xia:** Writing – original draft, Formal analysis. **Pingyu Wang:** Writing – original draft, Writing – review & editing. **Chao Xie:** Writing – review & editing, Investigation. **Hong-Li Lin:** Writing – review & editing. **Gloria Hoi-Yee Li:** Writing – review & editing, Investigation. **Cheng-Da Yuan:** Writing – review & editing, Investigation. **Mo-Chang Qiu:** Writing – review & editing, Validation. **Yi-Hu Fang:** Writing – review & editing, Validation. **Chun-Fu Yu:** Writing – review & editing, Validation. **Xiang-Chun Cai:** Software, Formal analysis. **Saber Khederzadeh:** Software, Formal analysis. **Pian-Pian Zhao:** Software, Formal analysis. **Meng-Yuan Yang:** Software, Formal analysis. **Jia-Dong Zhong:** Writing – review & editing, Methodology. **Xin Li:** Writing – review & editing, Methodology. **Peng-Lin Guan:** Writing – review & editing, Methodology. **Jia-Xuan Gu:** Writing – review & editing, Methodology. **Si-Rui Gai:** Methodology, Visualization. **Xiang-Jiao Yi:** Methodology, Visualization. **Jian-Guo Tao:** Methodology, Software. **Xiang Chen:** Methodology, Software. **Mao-Mao Miao:** Methodology, Visualization. **Guo-Bo Chen:** Data curation, Project administration. **Lin Xu:** Writing – review & editing. **Shu-Yang Xie:** Writing – review & editing. **Geng Tian:** Writing – review & editing. **Hua Yue:** Methodology, Visualization. **Guangfei Li:** Methodology, Visualization. **Wenjin Xiao:** Software, Visualization. **David Karasik:** Writing – review & editing, Methodology. **Youjia Xu:** Writing – review & editing, Investigation. **Liu Yang:** Writing – review & editing, Investigation. **Ching-Lung Cheung:** Writing – review & editing, Investigation. **Fei Huang:** Writing – review & editing. **Zhenlin Zhang:** Writing – review & editing, Investigation. **Hou-Feng Zheng:** Writing – review & editing, Data curation, Project administration, Supervision, Conceptualization, Funding acquisition. All authors have read and approved the final manuscript.

## ORCID

0000-0001-6754-5871 (Yu Qian)

0009-0006-6045-038X (Jiangwei Xia)

0000-0003-0980-4294 (Pingyu Wang)

0000-0002-9137-3835 (Chao Xie)

0009-0008-8104-4035 (Hong-Li Lin)

0000-0003-0275-2356 (Gloria Hoi-Yee Li)

0009-0001-0009-812X (Cheng-Da Yuan)

0000-0001-9468-8034 (Mo-Chang Qiu)

0009-0008-3624-2155 (Yi-Hu Fang)

0009-0009-7611-3558 (Chun-Fu Yu)

0009-0004-1547-1515 (Xiang-Chun Cai)

0000-0002-0115-8710 (Saber Khederzadeh)

0009-0009-1676-1748 (Pian-Pian Zhao)

0009-0001-3184-5706 (Meng-Yuan Yang)

0000-0003-4882-6488 (Jia-Dong Zhong)

0000-0002-8986-7887 (Xin Li)

0009-0002-5603-2012 (Peng-Lin Guan)

0009-0009-0443-3288 (Jia-Xuan Gu)

0009-0004-9166-7026 (Si-Rui Gai)

0000-0002-3782-3954 (Xiang-Jiao Yi)

0000-0002-3473-9212 (Jian-Guo Tao)

0000-0003-3188-8332 (Xiang Chen)

0009-0004-2909-7333 (Mao-Mao Miao)

0000-0001-5475-8237 (Guo-Bo Chen)

0009-0003-0610-9824 (Lin Xu)

0000-0002-8090-2180 (Shu-Yang Xie)

0000-0001-5877-5560 (Geng Tian)

0000-0002-9179-5894 (Hua Yue)

0000-0002-5527-8996 (Guangfei Li)

0000-0002-4922-385X (Wenjin Xiao)

0000-0002-8826-0530 (David Karasik)

0000-0002-9826-4324 (Youjia Xu)

0000-0002-6498-4702 (Liu Yang)

0000-0002-6233-9144 (Ching-Lung Cheung)

0000-0002-2298-4720 (Fei Huang)

0000-0002-4339-1617 (Zhenlin Zhang)

0000-0001-5681-8598 (Hou-Feng Zheng)
