# Supplementary material for: Genome-wide Association Studies of over 30,000 Samples with Bone Mineral Density at Multiple Skeletal Sites and Its Clinical Relevance
Source: Genomics Proteomics Bioinformatics. 2025 Nov 5;23(5):qzaf097. doi: 10.1093/gpbjnl/qzaf097 (PMC12996891; doi:10.1093/gpbjnl/qzaf097)
Supplement: qzaf097_Supplementary_Data [file qzaf097_supplementary_data.zip › File S1 103025.docx]

**File S1 Supplementary methods**

### Gene functional enrichment analysis

For the detected genes, we used KOBAS 3.0 to evaluate the underlying biological mechanisms by using gene ontology (GO) term enrichment analyses [1]. Statistical significance for enrichment analyses was set at an FDR-corrected P-value of 0.05.

### MR analysis

We have conducted a comprehensive MR analysis to assess the association between potential genetically predicted expression levels of candidate genes and BMD. First, we utilized the summary-based Mendelian randomization (SMR), under the assumption of either causality (where the effect of a genetic variant on a trait is mediated by gene expression) or pleiotropy (where a genetic variant has direct effects on both a trait and gene expression) [2]. The cis-eQTLs showing associations of *P* value <5×10^-8^ in the original tissue were selected as instrumental variables. Linkage clumping was also conducted according to default SMR protocols (--ld-upper-limit 0.9 --ld-lower-limit 0.05 --peqtl-heidi 1.57e-3 --heidi-min-m 3 --heidi-max-m 20 --cis-wind 2000).

For the associations supported by SMR evidence, we conducted additional MR analyses, including weighted median, maximum likelihood, weighted mode, and MR-Egger regression, to evaluate the robustness of these findings. The weighted median method showed that the results were reliable when more than 50% of the weights came from valid IVs [3,4]. The likelihood-based method assumes a bivariate normal distribution for the genetic associations with the risk factor and outcome [5]. The weighted mode-based estimators consistently estimated the causal effect when the mode across instruments of the horizontal pleiotropy effects was zero [6]. Furthermore, Under the assumption that the association of each genetic variant with the exposure is independent of the pleiotropic effect of the variant (not via the exposure), Egger’s test gives a valid test of the null causal hypothesis [7]. When the intercept of MR-Egger regression was not statistically different from 0 (P>0.05), it was considered that there was no horizontal pleiotropy [7].

Specifically, in these additional MR methods, for seven genes with SMR evidence (*i.e.*, ABCA1, CCR1, ESR1, NCOR1, PAX1, SREBF1, SWAP70), we selected the genome-wide significant independent cis-eQTL as instrumental variables to proxy the expression of corresponding genes. These selections were performed using PLINK 1.90 [8] with the parameters --clump-p1 5e-08, --clump-kb 250, --clump-r2 0.001), based on the corresponding cis-eQTL dataset from tissues from GTEx version 8 projects (artery tibial tissue) [9] and eQTLGen consortium (whole blood) [10]. A total of 5 cis-eQTL were selected as instrumental variables for ABCA1 gene expression (from whole blood tissue), 6 for SWAP70 gene expression (from whole blood tissue),4 for NCOR1 gene expression (from whole blood tissue), 8 for CCR1 gene expression (from whole blood tissue), 5 for SREBF1 gene expression (from whole blood tissue), 1 for PAX1 gene expression (from muscle skeletal tissue), and 1 for ESR1 gene expression (from whole blood tissue). When more than 3 instruments were available, we performed the weighted median, maximum likelihood, weighted mode, and MR-Egger regression to replicate SMR estimate associations.

Based on the SWAP70 pQTL dataset from deCODE, we selected the genome-wide significant independent SNPs across the whole genome as instrumental variables for circulating SWAP70, using PLINK1.90 software (--clump-p1 5e-08, --clump-kb 250, --clump-r2 0.001) [11]. After extraction of genetic association estimates for exposure (*i.e.*, circulating SWAP70) and outcome (*i.e.*, BMD), we performed the inverse-variance weighted method and supplied the weighted median method to assess the association between genetically predicted circulating SWAP70 and BMD.

Finally, we also assessed the bidirectional MR analyses and sensitivity analyses to assess the potential causal association between head BMD and IA. Specifically, for the analysis of the association of head BMD with IA, we selected instrumental variables using PLINK 1.90 based on the same parameters (*i.e.*, --clump-p1 5e-08, --clump-kb 250, and --clump-r2 0.001). A total of 56 SNPs were included as instrumental variables for head BMD. For the reverse direction, we evaluated the effect of IA on head BMD. A total of 10 SNPs were selected as instrumental variables for IA using the same selection criteria. We performed the inverse-variance weighted method and supplied the weighted median method, maximum likelihood method, and MR-Egger regression to assess the bidirectional association between head BMD and IA.

**References**

[1] Bu D, Luo H, Huo P, Wang Z, Zhang S, He Z, et al. KOBAS-i: Intelligent prioritization and exploratory visualization of biological functions for gene enrichment analysis. Nucleic Acids Res 2021;49:W317–25.

[2] Zhu Z, Zhang F, Hu H, Bakshi A, Robinson MR, Powell JE, et al. Integration of summary data from GWAS and eQTL studies predicts complex trait gene targets. Nat Genet 2016;48:481–7.

[3] Liu B, Lyu L, Zhou W, Song J, Ye D, Mao Y, et al. Associations of the circulating levels of cytokines with risk of amyotrophic lateral sclerosis: A mendelian randomization study. BMC Med 2023;21:39.

[4] Bowden J, Davey Smith G, Haycock PC, Burgess S. Consistent estimation in mendelian randomization with some invalid instruments using a weighted median estimator. Genet Epidemiol 2016;40:304–14.

[5] Burgess S, Butterworth A, Thompson SG. Mendelian randomization analysis with multiple genetic variants using summarized data. Genet Epidemiol 2013;37:658–65.

[6] Hartwig FP, Davey Smith G, Bowden J. Robust inference in summary data mendelian randomization via the zero modal pleiotropy assumption. Int J Epidemiol 2017;46:1985–98.

[7] Burgess S, Thompson SG. Interpreting findings from mendelian randomization using the MR-egger method. Eur J Epidemiol 2017;32:377–89.

[8] Chang CC, Chow CC, Tellier LC, Vattikuti S, Purcell SM, Lee JJ. Second-generation PLINK: Rising to the challenge of larger and richer datasets. Gigascience 2015;4:7.

[9] Võsa U, Claringbould A, Westra H-J, Bonder MJ, Deelen P, Zeng B, et al. Large-scale cis- and trans-eQTL analyses identify thousands of genetic loci and polygenic scores that regulate blood gene expression. Nat Genet 2021;53:1300–10.

[10] GTEx Consortium. Human genomics. The genotype-tissue expression (GTEx) pilot analysis: Multitissue gene regulation in humans. Science 2015;348:648–60.

[11] Ferkingstad E, Sulem P, Atlason BA, Sveinbjornsson G, Magnusson MI, Styrmisdottir EL, et al. Large-scale integration of the plasma proteome with genetics and disease. Nat Genet 2021;53:1712–21.
