# Supplementary material for: Genome-wide Association Studies of over 30,000 Samples with Bone Mineral Density at Multiple Skeletal Sites and Its Clinical Relevance
Source: Genomics Proteomics Bioinformatics. 2025 Nov 5;23(5):qzaf097. doi: 10.1093/gpbjnl/qzaf097 (PMC12996891; doi:10.1093/gpbjnl/qzaf097)
Supplement: qzaf097_Supplementary_Data [file qzaf097_supplementary_data.zip › supplementary material captions.docx]

**Supplementary materials**

**File S1 Supplementary methods**

**Figure S1 The identified loci for the 11 BMD sites and fracture**

**Figure S2 Manhattan and quantile-quantile plots for arm BMD**

**Figure S3 Manhattan and quantile-quantile plots for femoral neck BMD**

**Figure S4 Manhattan and quantile-quantile plots for femur total BMD**

**Figure S5 Manhattan and quantile-quantile plots for head BMD**

**Figure S6 Manhattan and quantile-quantile plots for leg BMD**

**Figure S7 Manhattan and quantile-quantile plots for lumbar spine BMD**

**Figure S8 Manhattan and quantile-quantile plots for pelvis BMD**

**Figure S9 Manhattan and quantile-quantile plots for rib BMD**

**Figure S10 Manhattan and quantile-quantile plots for spine BMD**

**Figure S11 Manhattan and quantile-quantile plots for trunk BMD**

**Figure S12 Manhattan and quantile-quantile plots for total BMD**

**Figure S13 Manhattan and quantile-quantile plots for any type of bone fracture**

**Figure S14 Regional association plots of flanking 250kb region around the rs746100, based on femur total BMD GWAS and *ABCA1* eQTL**

The x-axis denotes the physical position of each genetic variant on the chromosome specified, whereas the y-axis indicates the evidence of association, which was shown as -log_10_(*P*-value). Abbreviations: BMD, bone mineral density; GWAS, genome-wide association study; eQTL, expression quantitative trait locus.

**Figure S15**  **Regional association plots of flanking 250kb region around the rs10840273, based leg BMD GWAS, *SWAP70* eQTL, and *SWAP70* pQTL**

The x-axis denotes the physical position of each genetic variant on the chromosome specified, whereas the y-axis indicates the evidence of association, which was shown as -log_10_(*P*-value). Abbreviations: BMD, bone mineral density; GWAS, genome-wide association study; eQTL, expression quantitative trait locus; pQTL, protein quantitative trait locus.

**Figure S16 Locuszoom of rs12916774 for femoral neck BMD**

**Figure S17 Locuszoom of rs6013897 for femoral neck BMD**

**Figure S18 Locuszoom of rs927059 for femoral neck BMD**

**Figure S19 Manhattan and quantile-quantile plots for weight-bearing bone fracture**

**Figure S20 Manhattan and quantile-quantile plots for other bone fracture**

**Figure S21 Cumulative incidence curves for incident fracture across polygenic risk categories in the whole population**

**Table S1 The result from LDSC regression.**

**Table S2 Detailed information on 240 unique conditional independent BMD signals**

**Table S3 The positional annotation results for BMD-related independent SNP**

**Table S4 The eQTL annotation results for BMD-related independent SNP**

**Table S5 The result of MR methods**

**Table S6 The HiC annotation results for BMD-related independent SNP**

**Table S7 The OMIM annotation results for BMD-related independent SNP**

**Table S8 Sex-stratified GWAS results**

**Table S9 The detailed information from PRSice-2 software**

**Table S10 The result from stepwise Cox regression**

**Table S11 The result of the association between metaPRS and fracture risk**

**Table S12 The LDSC result between DXA-BMD and 13 common chronic diseases**

**Table S13 The result of MixeR analyses**

**Table S14 The result of the conjFDR method**

**Table S15 The colocalization results of four pleiotropic loci**

**Table S16 The enrichment result of pleiotropic genes**

**Table S17 Bidirectional MR results for the association between head BMD and IA**

**Table S18 The colocalization results of 15 candidate genes**

**Table S19 Results from the SharedPro method**

**Table S20 Detailed information on the field ID and code for fracture case definition**

**Table S21 The whole druggable genome from ChEMBL v29 datasets**
