# Supplementary figures and images for: Genome-wide Association Studies of over 30,000 Samples with Bone Mineral Density at Multiple Skeletal Sites and Its Clinical Relevance
Source: Genomics Proteomics Bioinformatics. 2025 Nov 5;23(5):qzaf097. doi: 10.1093/gpbjnl/qzaf097 (PMC12996891; doi:10.1093/gpbjnl/qzaf097)

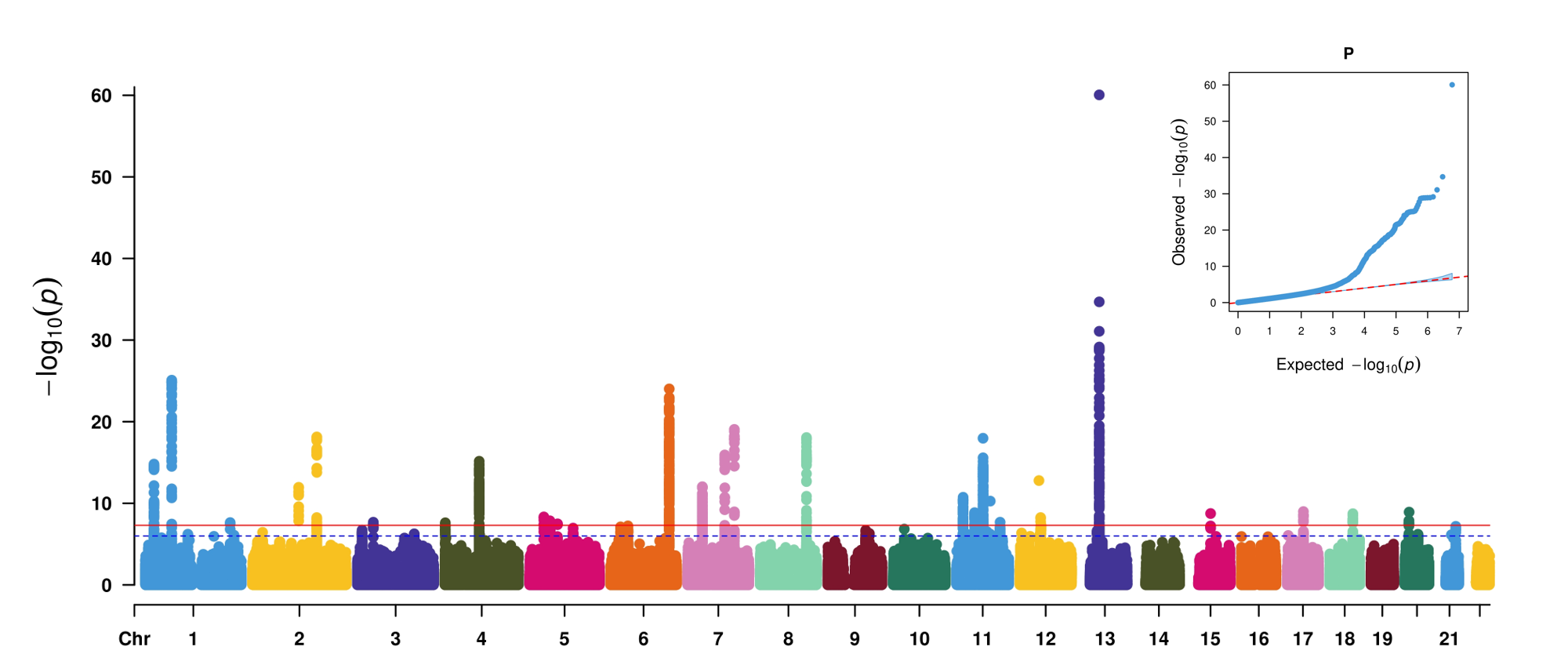

Supplement: qzaf097_Supplementary_Data [file qzaf097_supplementary_data.zip › Figure_S10.png]

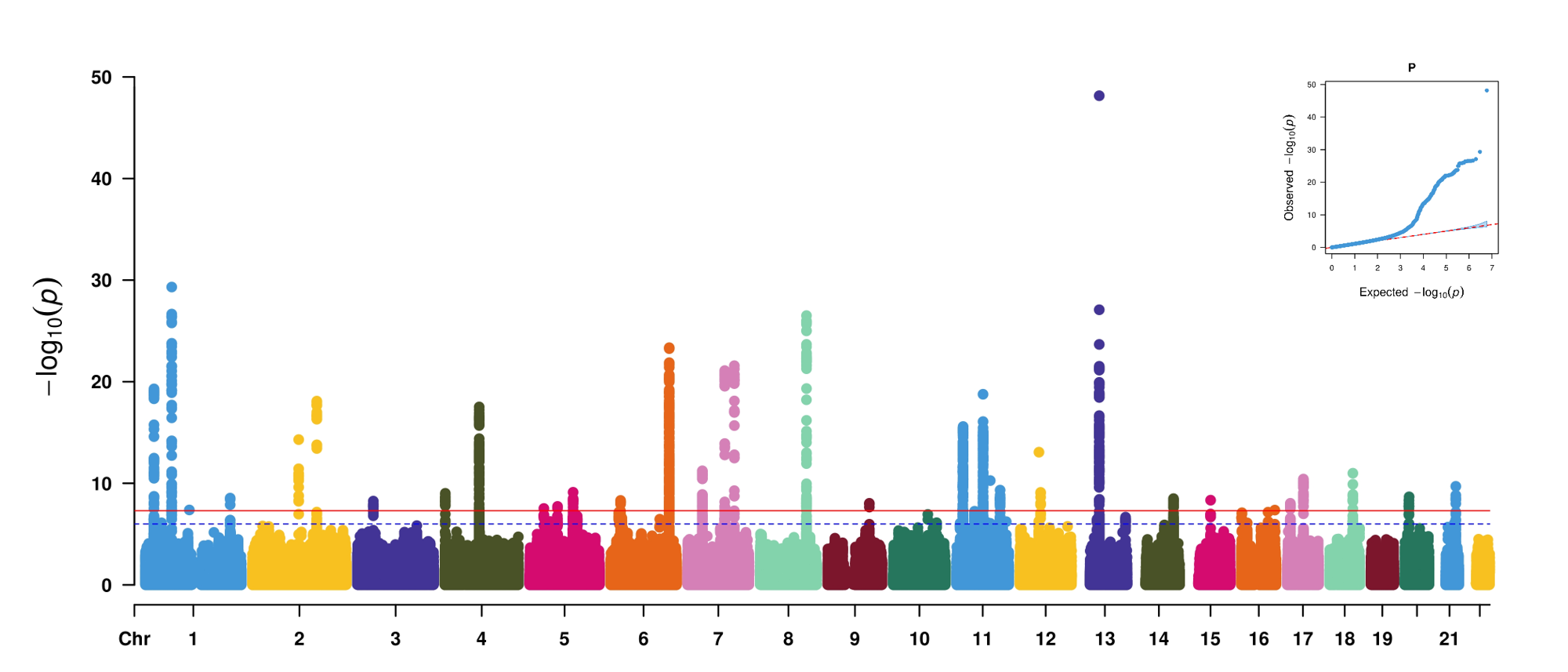

Supplement: qzaf097_Supplementary_Data [file qzaf097_supplementary_data.zip › Figure_S11.png]

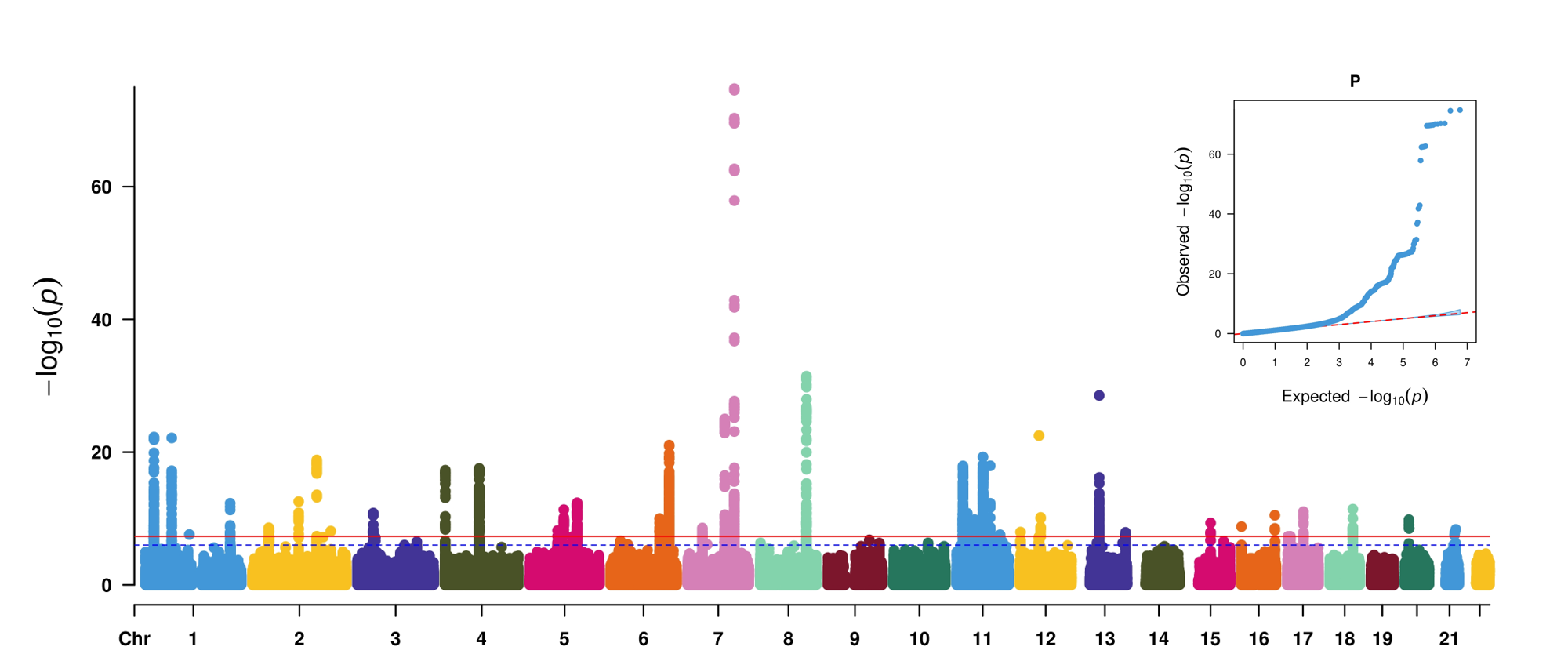

Supplement: qzaf097_Supplementary_Data [file qzaf097_supplementary_data.zip › Figure_S12.png]

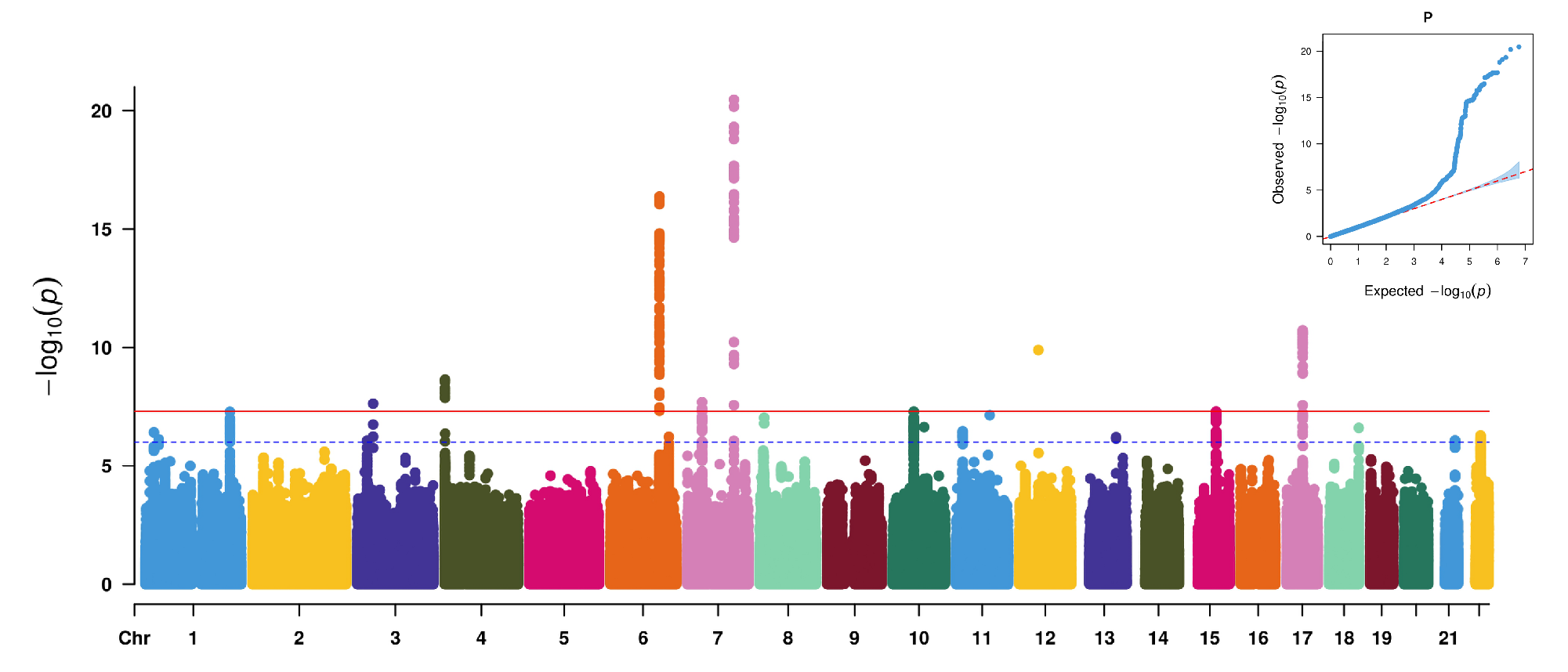

Supplement: qzaf097_Supplementary_Data [file qzaf097_supplementary_data.zip › Figure_S13.png]

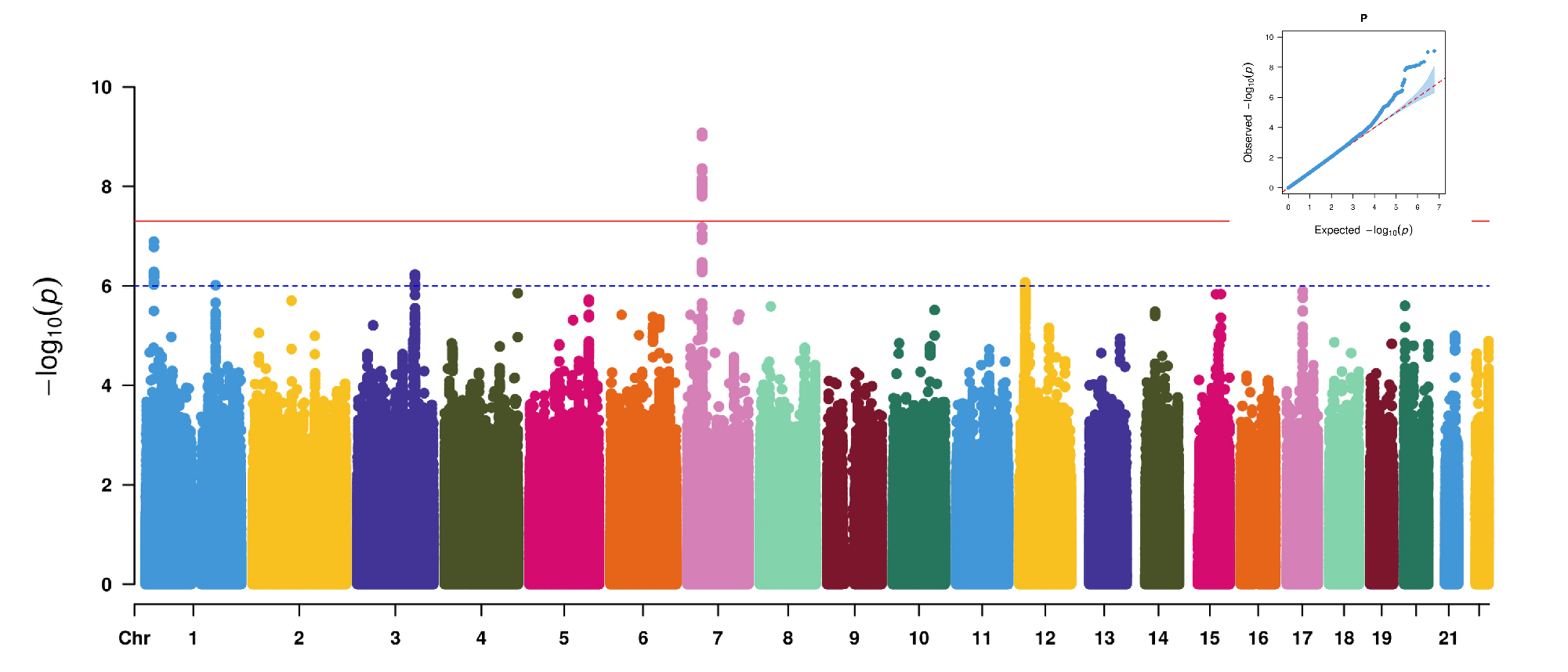

Supplement: qzaf097_Supplementary_Data [file qzaf097_supplementary_data.zip › Figure_S19.png]

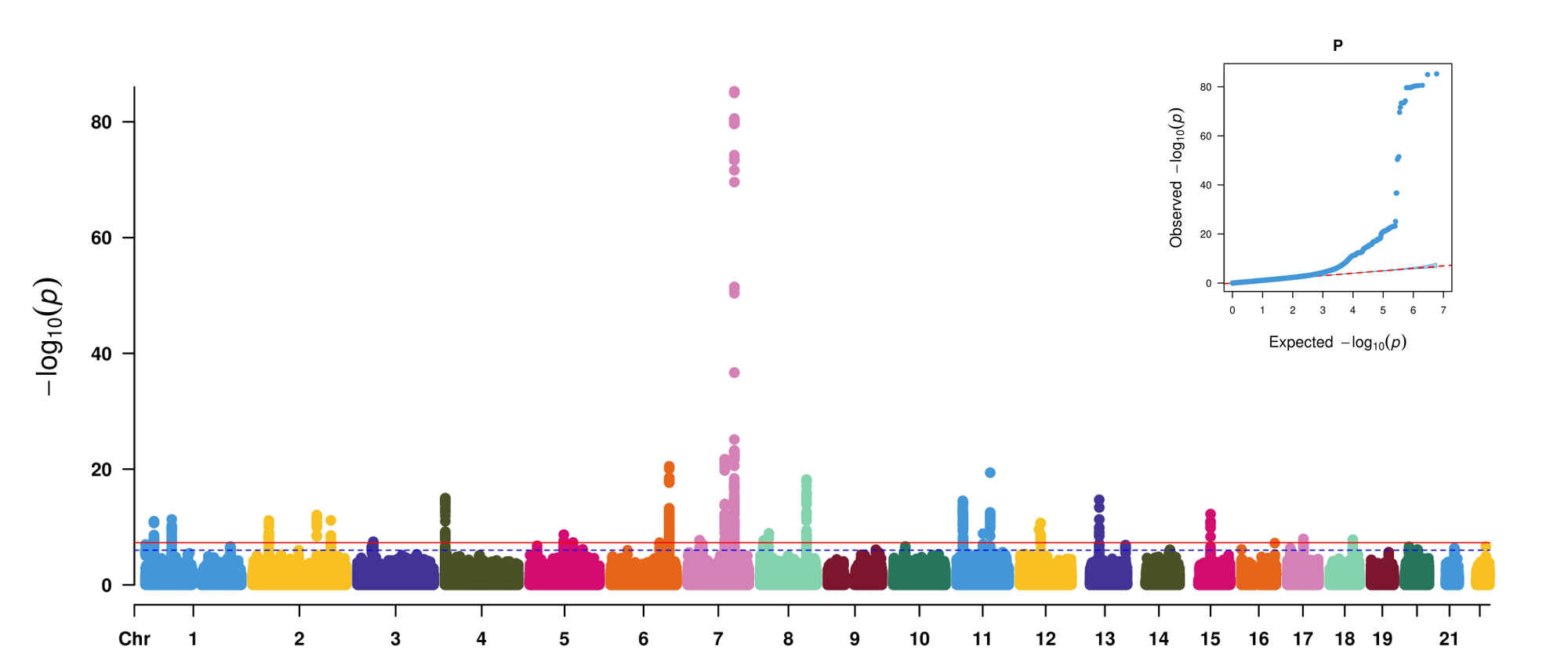

Supplement: qzaf097_Supplementary_Data [file qzaf097_supplementary_data.zip › Figure_S2.png]

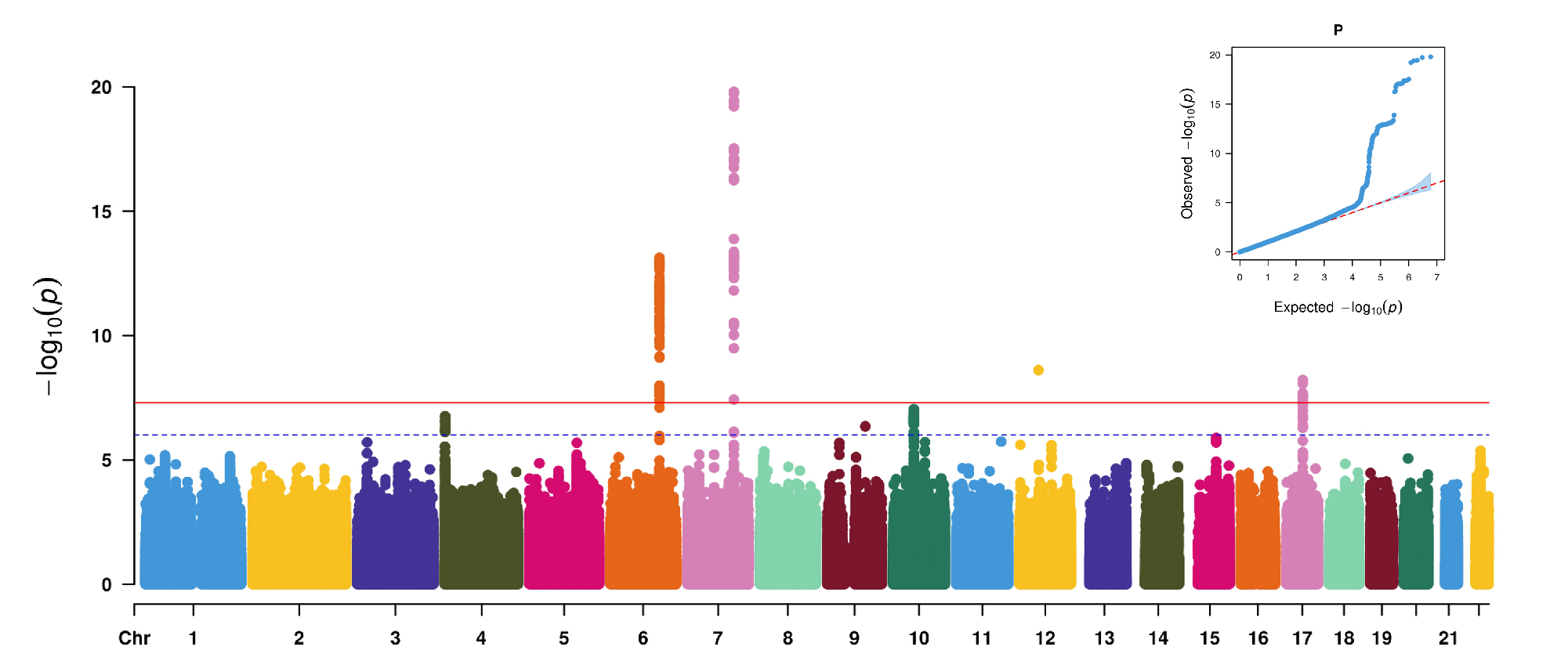

Supplement: qzaf097_Supplementary_Data [file qzaf097_supplementary_data.zip › Figure_S20.png]

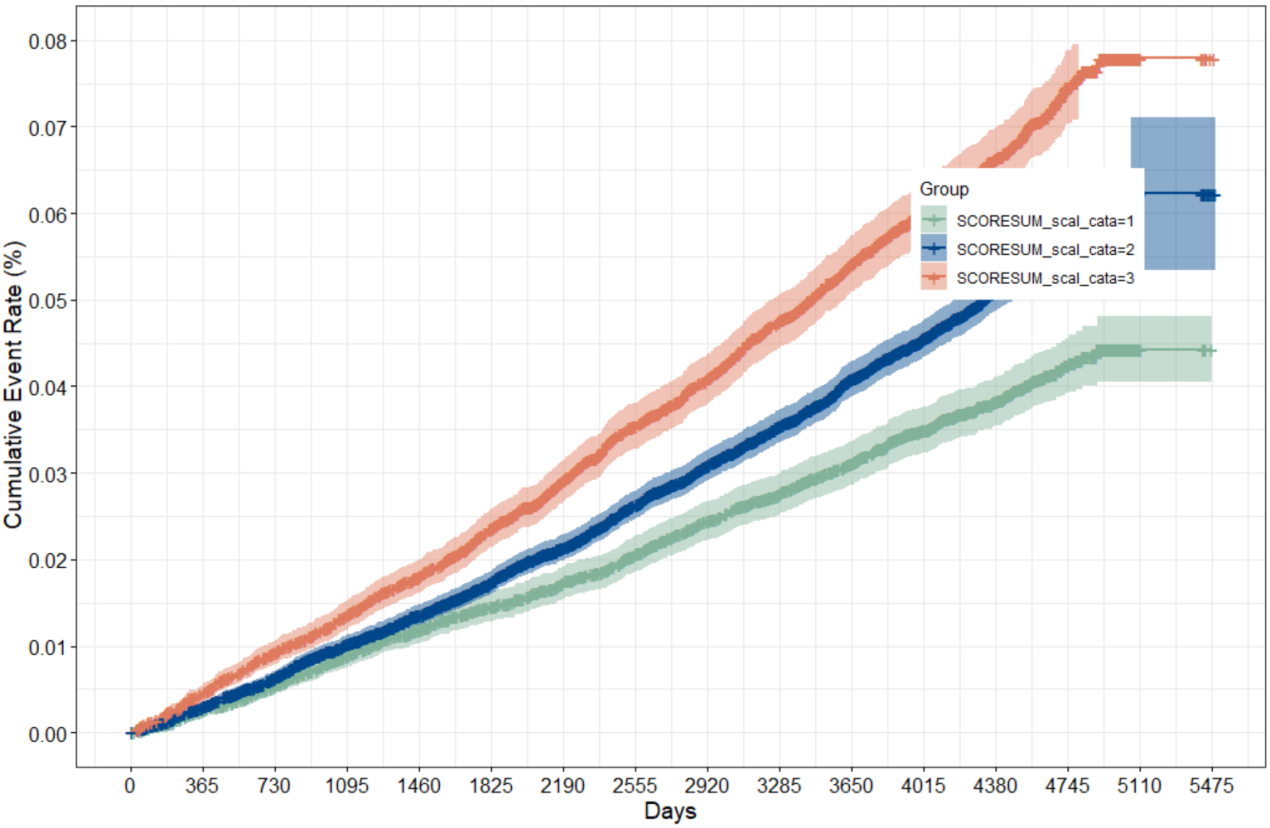

Supplement: qzaf097_Supplementary_Data [file qzaf097_supplementary_data.zip › Figure_S21.png]

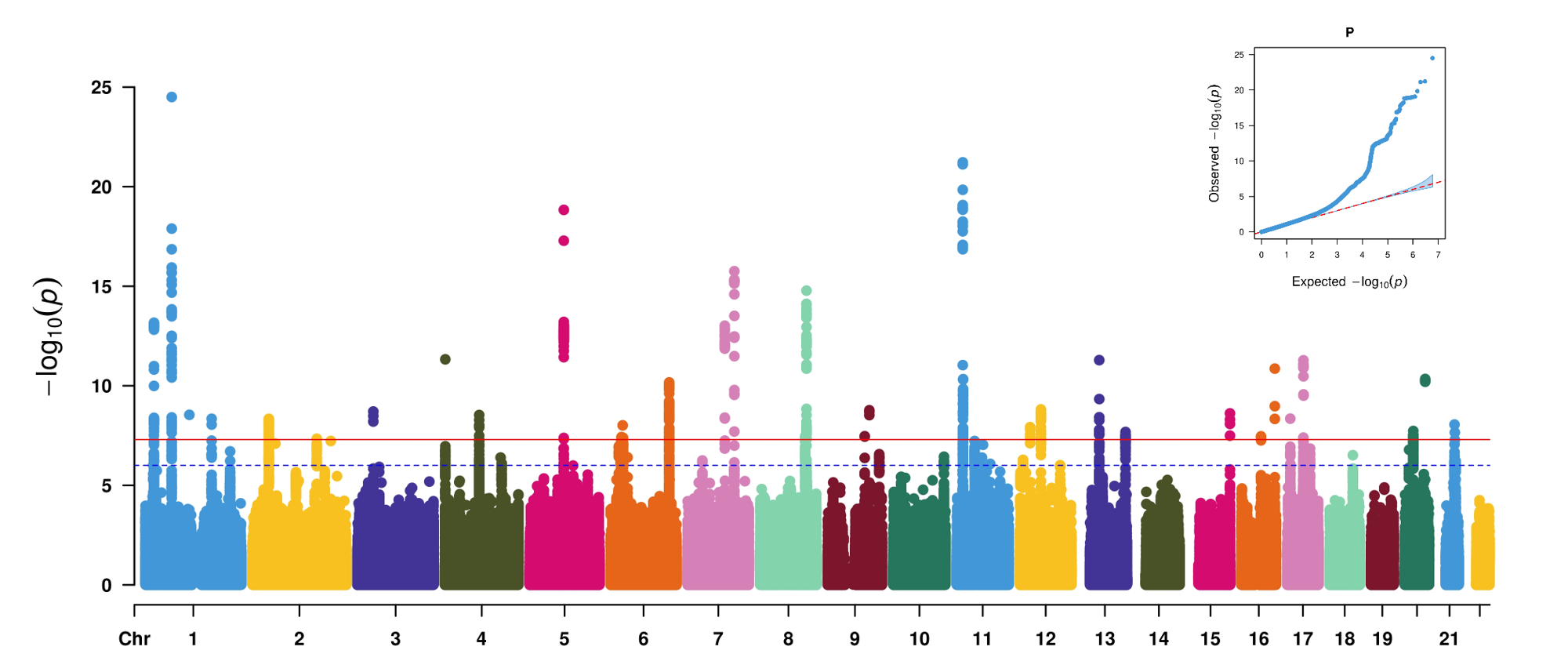

Supplement: qzaf097_Supplementary_Data [file qzaf097_supplementary_data.zip › Figure_S3.png]

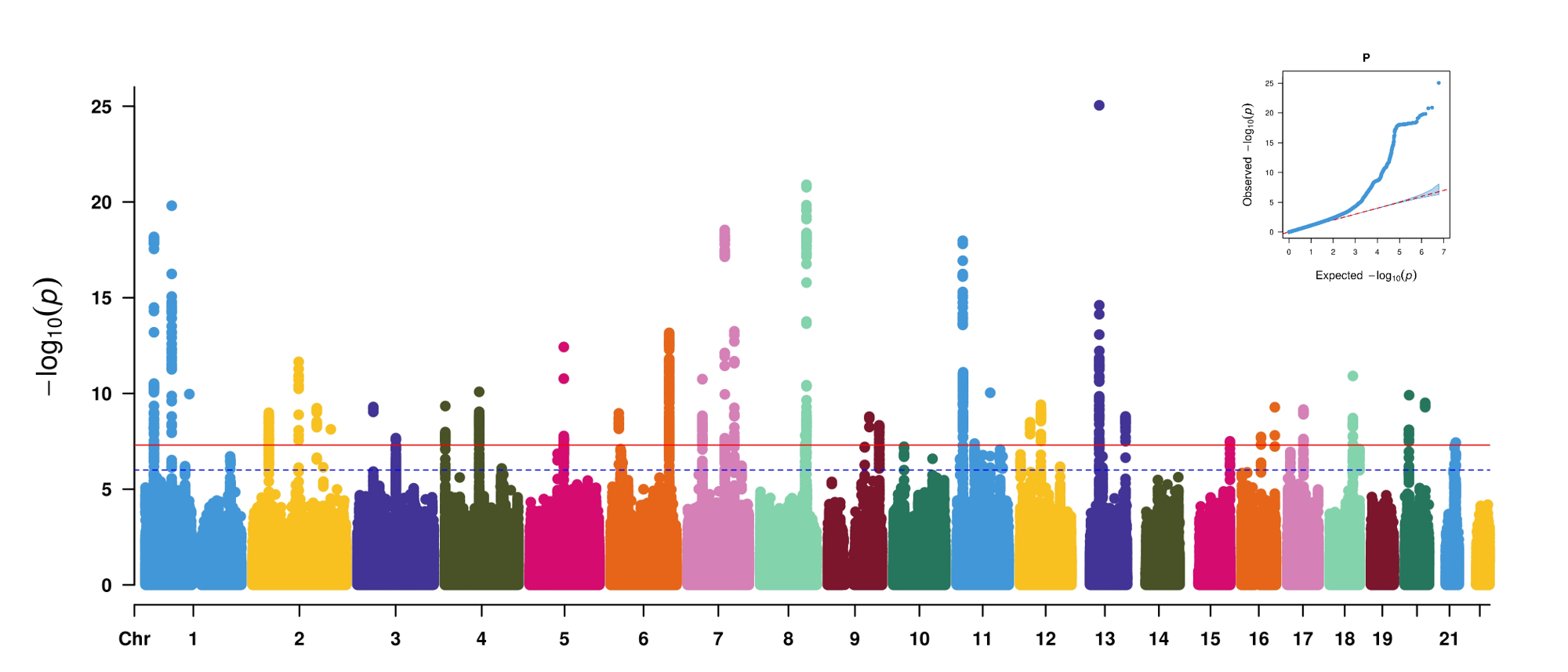

Supplement: qzaf097_Supplementary_Data [file qzaf097_supplementary_data.zip › Figure_S4.png]

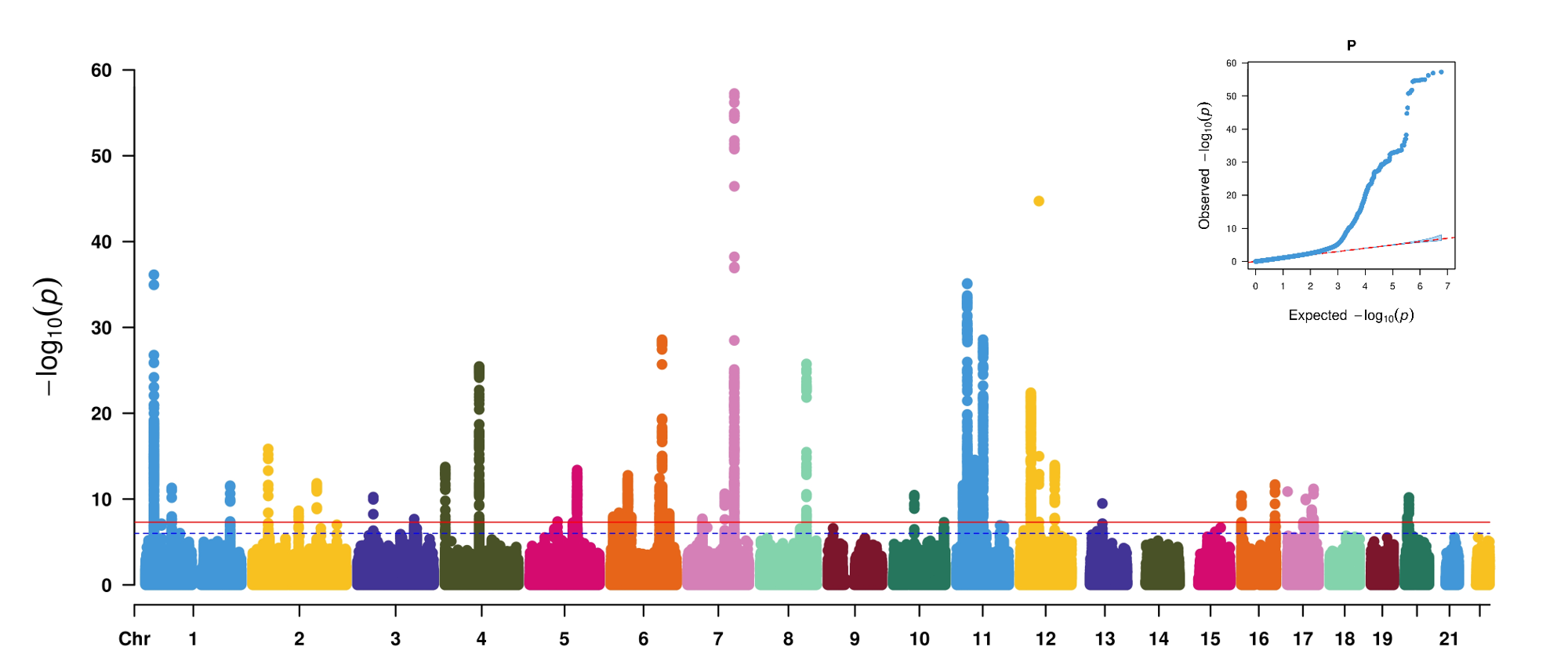

Supplement: qzaf097_Supplementary_Data [file qzaf097_supplementary_data.zip › Figure_S5.png]

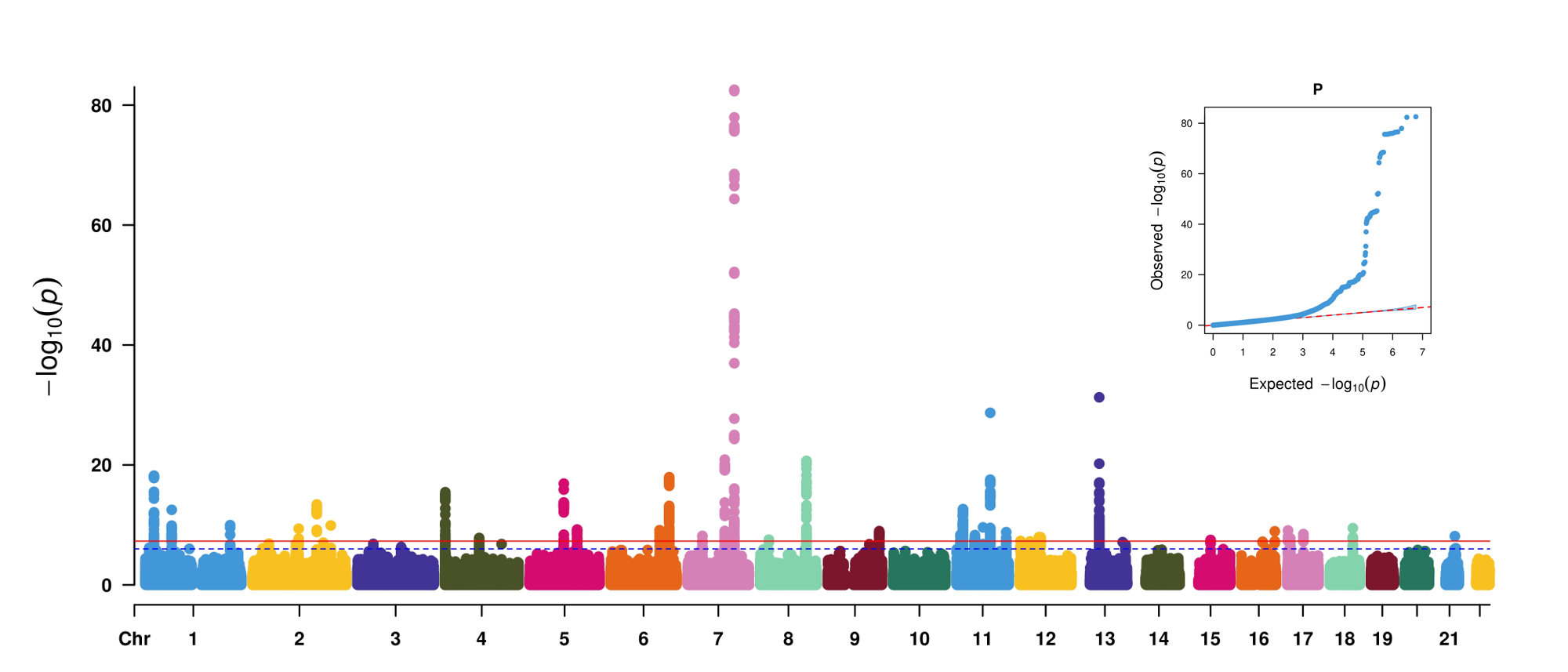

Supplement: qzaf097_Supplementary_Data [file qzaf097_supplementary_data.zip › Figure_S6.png]

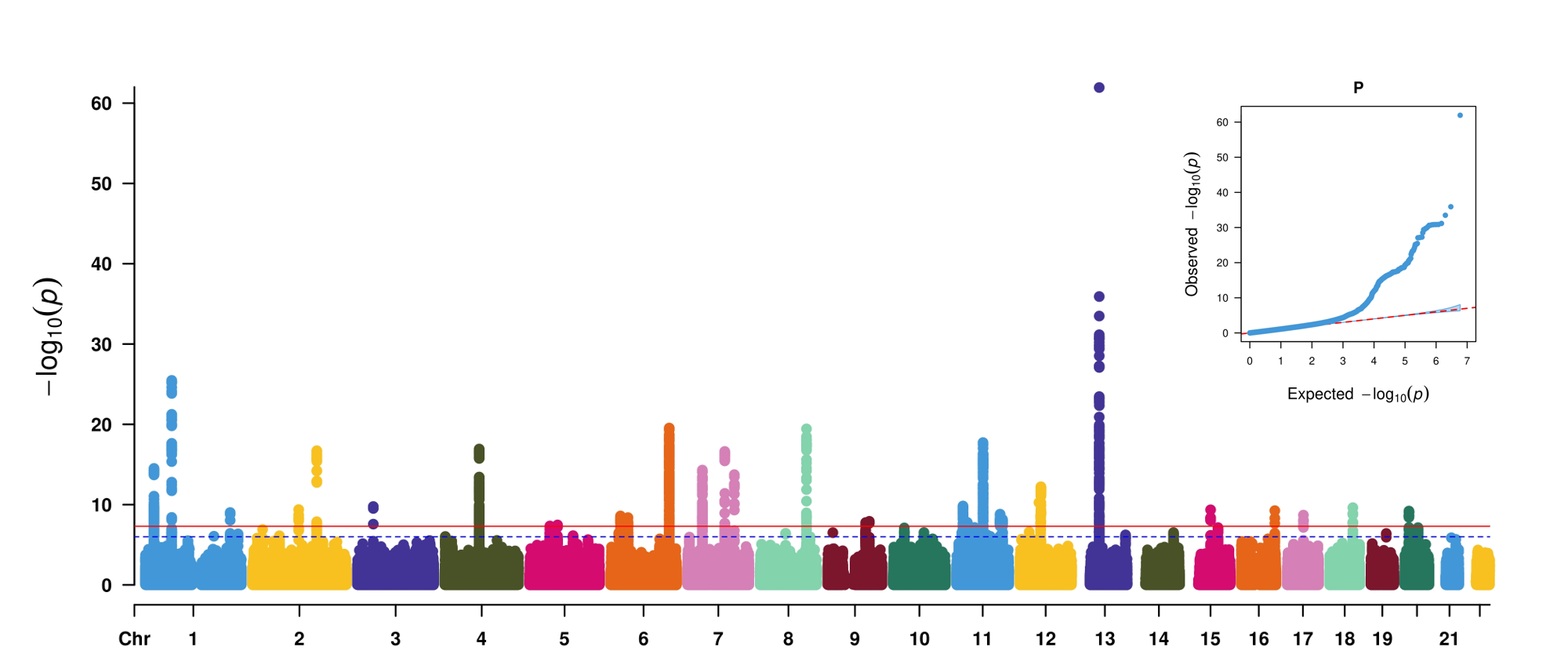

Supplement: qzaf097_Supplementary_Data [file qzaf097_supplementary_data.zip › Figure_S7.png]

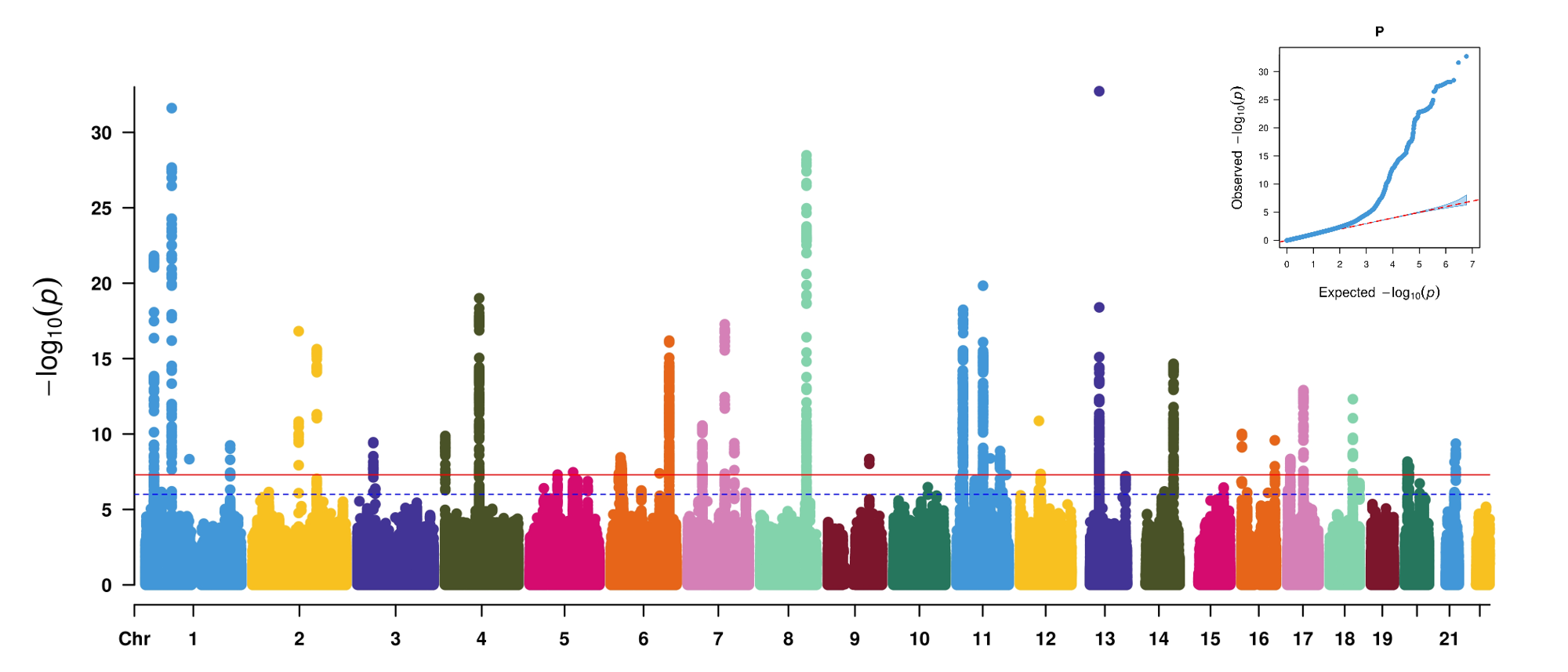

Supplement: qzaf097_Supplementary_Data [file qzaf097_supplementary_data.zip › Figure_S8.png]

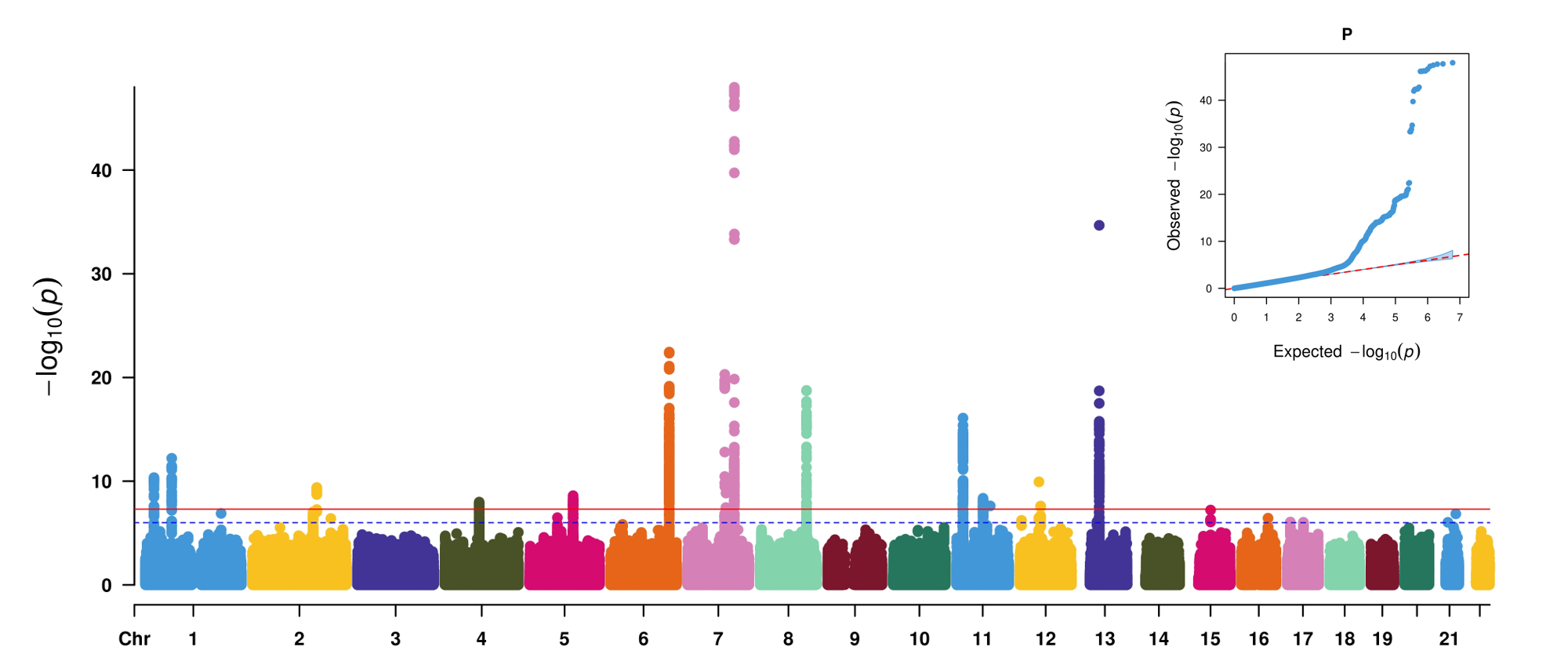

Supplement: qzaf097_Supplementary_Data [file qzaf097_supplementary_data.zip › Figure_S9.png]
